# C/D-box snoRNA-derived RNA production is associated with malignant transformation and metastatic progression in prostate cancer

**DOI:** 10.18632/oncotarget.4172

**Published:** 2015-05-19

**Authors:** Elena S. Martens-Uzunova, Youri Hoogstrate, Anton Kalsbeek, Bas Pigmans, Mirella Vredenbregt-van den Berg, Natasja Dits, Søren Jensby Nielsen, Adam Baker, Tapio Visakorpi, Chris Bangma, Guido Jenster

**Affiliations:** ^1^ Department of Urology, Erasmus MC, Rotterdam, The Netherlands; ^2^ Exiqon A/S, Vedbaek, Denmark; ^3^ Nuevolution A/S, Copenhagen, Denmark; ^4^ Chr. Hansen A/S, Hørsholm, Denmark; ^5^ Institute of Biosciences and Medical Technology - BioMediTech, University of Tampere and Tampere University Hospital, Tampere, Finland

**Keywords:** snoRNA, sdRNA, SNORD78, GAS5, prostate cancer

## Abstract

Small nucleolar RNAs (snoRNAs) are dynamically regulated in different tissues and affected in disease. SnoRNAs are processed further to stable smaller RNAs. We sequenced the small RNA transcriptome of prostate cancer (PCa) at different PCa stages and generated a quantified catalogue of 3927 small non-coding RNAs (sncRNAs) detected in normal and malignant prostate tissue. From these, only 1524 are microRNAs. The remaining 2401 sncRNAs represent stable sncRNAs species that originate from snoRNA, tRNA and other sncRNAs. We show that snoRNA-derived RNAs (sdRNAs) display stronger differential expression than microRNAs and are massively upregulated in PCa. SdRNAs account for at least one third of all small RNAs with expression changes in tumor compared to normal adjacent tissue. Multiple sdRNAs can be produced from one snoRNA in a manner related to the conservation of structural snoRNA motifs. Q-PCR analysis in an independent patient cohort (n=106) confirmed the processing patterns of selected snoRNAs (*SNORD44, SNORD78, SNORD74* and *SNORD81*) and the cancer-associated up-regulation of their sdRNAs observed in sequencing data. Importantly, expression of *SNORD78* and its sdRNA is significantly higher in a subset of patients that developed metastatic disease demonstrating that snoRNA and sdRNAs may present as novel diagnostic and/or prognostic biomarkers for PCa.

## INTRODUCTION

Malignant transformation and cancer progression cause changes in the expression and function of microRNAs (miRNAs) [1, 2]. However, the effects of these processes on other small non-coding RNAs (sncRNAs) are less understood. Recently, we demonstrated the abundance and differential expression of small nucleolar RNA-derived RNAs (sdRNAs) in the small transcriptome of prostate cancer (PCa) [3]. It is generally accepted that small nucleolar RNAs (snoRNAs) are housekeeping, protein-noncoding molecules that associate with specific sets of proteins to maintain proper ribosomal maturation in the nucleolus.

Still, several reports show that snoRNAs have tissue-specific expression [4, 5], and may present as novel cancer biomarkers. For example, the H/ACA-box snoRNA SNORA42 is commonly overexpressed in non-small cell lung cancer (NSCLC) and its expression is significantly inversely correlated with survival [6, 7]. Similarly, the levels of C/D-box snoRNAs SNORD33, SNORD66 and SNORD76 are significantly elevated in plasma from NSCLC patients compared with cancer-free controls and can provide potential biomarkers for early detection [8]. In chronic lymphocytic leukemia (CLL), heterogeneous snoRNA expression patterns discriminate major CLL subgroups and can stratify patients in different prognostic groups [9], while in multiple myeloma snoRNA expression patterns are associated with distinct molecular subtypes of the disease [10].

Furthermore, resent research demonstrates that the molecular alterations of snoRNA are functionally linked to basic cellular processes associated with cancer proposing either tumor suppressor or oncogene role for different snoRNAs. In NSCLC, SNORA42 acts as a putative oncogene. Its overexpression enhances cell proliferation and growth in bronchial epithelium and cancer cells, while its knockdown in NSCLC cells inhibits colony forming [7]. In acute promyelocytic leukemia, the SNORD112–114 is specifically activated in a subset of patients and may influence cell growth through a negative regulation of the cell cycle and the Rb pathway [11]. On the contrary, in peripheral T-cell lymphoma, over-expression of the candidate prognostic marker SNORD71 (HBII-239) is associated with favorable outcome [12]. The C/D box snoRNA SNORD50, a translocation partner of BCL6 in B-cell lymphoma [13], is a candidate tumor suppressor significantly associated with clinically relevant prostate [14] and breast [15] cancer. In hepatocellular carcinoma (HCC), SNORD113-1 has been identified as a tumor suppressor [16]. Down-regulation of this snoRNA is associated with decreased survival of HCC patients, while reconstitution of its expression suppresses tumorigenesis *in vitro* and *in vivo*. In glioblastoma, decreased expression of the GAS5 encoded SNORD76 is associated with an aggressive phenotype [17]. Ectopic expression of this tumor suppressor snoRNA inhibits tumorigenicity by arresting cancer cells in S phase *in vitro* and inhibits orthotopic tumor growth *in vivo*. In breast cancer and head and neck squamous cell carcinoma the low expression of another GAS5 encoded snoRNA, SNORD44, correlates with markers of aggressive pathology and poor prognosis [18, 19].

At present, little is known about the pathways of snoRNA turnover. Apparently, snoRNAs are further processed to sdRNAs in a vast variety of organisms [20]. It is yet unclear whether sdRNAs are novel functional entities or footprint-products of snoRNA downstream processing shielded from degradation by snoRNA-interacting proteins. A miRNA-like activity has been proposed for sdRNAs derived from H/ACA-box snoRNAs (H/ACA-sdRNAs) based on their apparent size of 20-24 nt equivalent to miRNAs, the ability to promote repression of complementary targets *in vitro*, and the association with Dicer and AGO complexes [21-27]. In contrast, a bimodal size distribution of 17-20 nt and 27-30 nt has been reported for sdRNAs derived from C/D-box snoRNAs (C/D-sdRNAs) [3, 22, 23]. C/D-sdRNAs are not efficiently incorporated in AGO2 suggesting a different function for this type of sdRNAs [28]. In addition, it has been reported that the highly abundant in brain ‘orphan’ snoRNAs, SNORD115 and SNORD116, are processed into larger sdRNAs (34-73 nt) that complex with spliceosomal proteins and may regulate the alternative splicing of target mRNAs [29, 30]. Association of C/D-box snoRNAs with novel RNPs and involvement in alternative splicing has been previously observed in mice for the brain specific *MbII-52* [31]. Interestingly, both *MbII-52* and its human ortholog *SNORD115* produce larger sdRNAs (34-73 nt). Similar observation has also been made for sdRNA regions of *SNORD88C,* which can influence the alternative splicing of *FGFR3* pre-mRNA [32]. At the same time, studies in *Drosophila sp*. and in human cells show that snoRNAs are strongly enriched in the nuclear fractions of chromatin-associated RNA and possibly involved in the maintenance of open chromatin structure [33].

Here, we report the deep sequencing of patient-derived samples from normal prostate, and PCa in different disease stages, which reveals sdRNA production from the vast majority of known human snoRNAs. At least 78 of the detected sdRNAs demonstrate strong differential expression in cancer. Furthermore, the expression of some sdRNAs and their precursors is associated with clinical progression and metastatic occurrence.

## RESULTS

### Library preparation and sequencing

**Figure 1:**
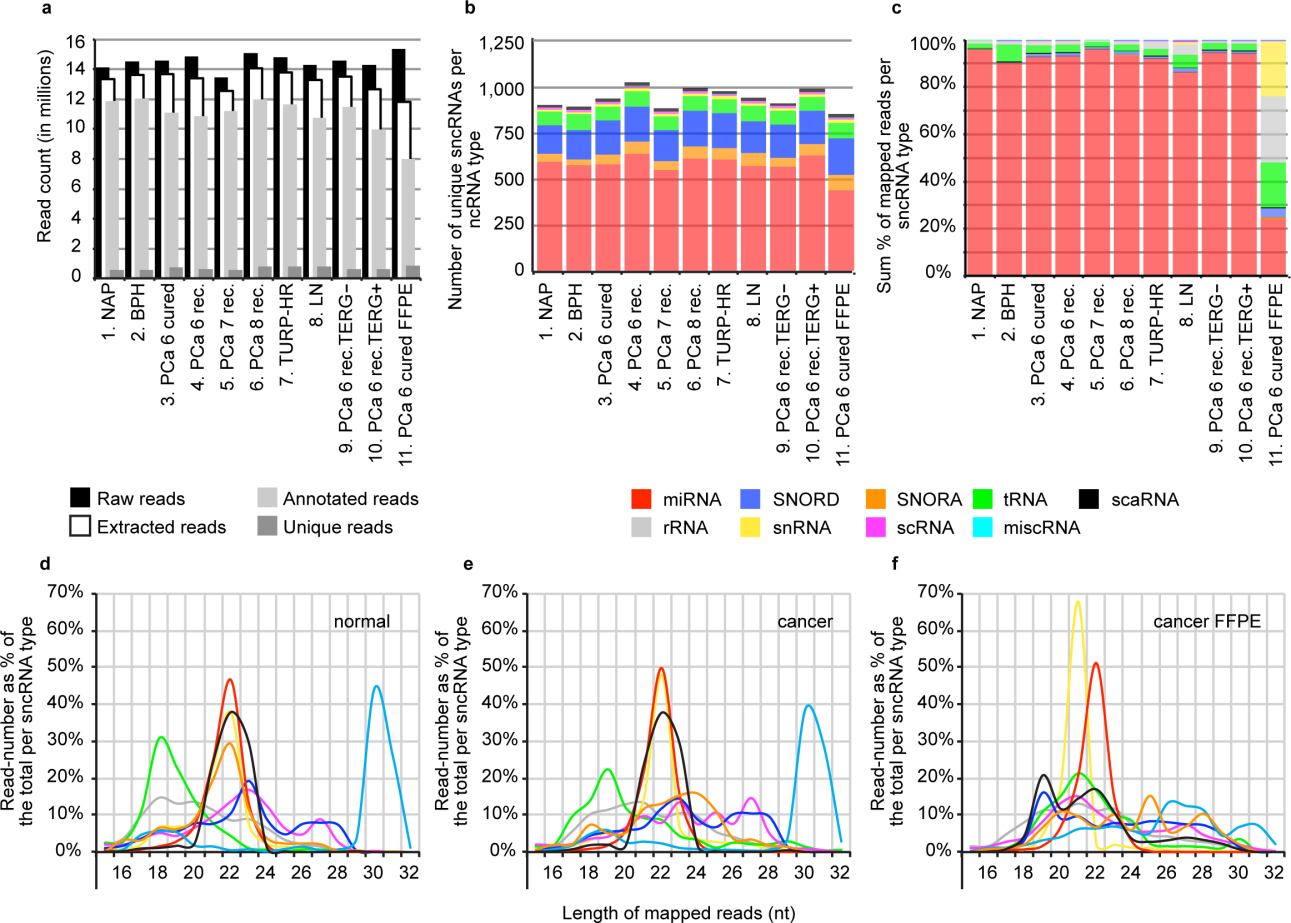
Summary of sncRNA sequencing data from PCa patient samples (**a**) Number of retrieved raw, extracted, annotated, and unique reads generated for each one of the sequencing libraries. (**b**) Number of detected sncRNA-species per library. (**c**) Relative abundance of different sncRNA-types per library. Read-length distribution in normal (**d**) and cancer libraries (**e**) derived from fresh-frozen, (**f**) and FFPE material. Each sncRNA type is represented by different color: miRNA (red), SNORD (dark blue), SNORA (orange), tRNA (green), scaRNA (black), rRNA (gray), snRNA (yellow), scRNA (magenta), other miscellaneous RNAs (light blue).

We generated 10 sncRNA libraries from normal adjacent prostate (NAP), benign prostate hyperplasia (BPH), different stages of PCa, and metastatic lymph node (LN) prepared from fresh-frozen patient material (FF) (Supplementary Table 1). To estimate the influence of sample storage on sncRNA abundance and stability, we prepared a replicate library from formalin-fixed, paraffin-embedded tissue (FFPE) from tumor samples used for one of the fresh-frozen libraries (group 3). All sequencing reactions yielded approximately 14 million raw reads each (13,468,284 to 15,393,670) with the FFPE library producing the highest raw read number (Figure 1a).

### Annotation of the sncRNA transcriptome

The correct mapping of sncRNA reads is challenged by the fact that predominant isoforms of miRNAs and other sncRNAs such as snoRNAs may vary from the mature sequences annotated in public databases. Differences can be caused by alternative 3′-end modifications [34] or alternative 5′-/3′-end positions of the detected sncRNA. Additionally, the length of mature sncRNA transcripts can be ambiguously annotated in different public databases. To map as many sequence reads as possible, we constructed a custom small non-coding RNA database (sncRNAdb) that consists of 2271 unique small non-coding RNA species corresponding to 2356 unique genomic loci (Supplementary Figure 1 and Supplementary File 1).

Mapping to sncRNAdb resulted in the detection of a total of 1637 unique sncRNAs expressed across any of the 11 libraries with an average of 1229 per library. 70% to 84% of the reads generated from fresh-frozen samples and only 52% of the reads generated from FFPE could be annotated by sncRNAdb (Figure 1a and Supplementary Table 2). The majority of annotated reads mapped to 873 pre-miRNAs (85.5 - 95.6%), 385 tRNAs (1.89 - 7.4%), 228 C/D-box snoRNAs (0.3 - 1.9%), and 91 H/ACA-box snoRNAs (0.0 - 0.1%) (Figure 1b, 1c and Supplementary Tables 2 and 3).

Interestingly, in PCa samples we detected up to 27% more C/D-box and up to 52% more H/ACA-box snoRNAs compared to NAP or BPH. Furthermore, total snoRNA read-counts were increased at least two-fold, indicating possible activation of snoRNA-gene expression in response to malignant transformation. In contrast, the number of detected miRNAs remained relatively stable and the total miRNA read-counts changed by no more than 19% (min. 9,202,300, max. 11,367,682) (Figure 1c, Supplementary Figure 2 and Supplementary Tables 3 and 4).

We also examined the read-length associated with different types of sncRNAs. As expected, miRNA reads had a narrow size distribution between 21 and 23 nt in all libraries. Similar size range was observed for snRNA- and scaRNA-derived RNAs in fresh-frozen libraries. In concordance with our previous results [3], we detected a size peak at 23 nt and a plateau between 26-28 nt for reads mapping to C/D-box snoRNAs. Interestingly, reads mapping to H/ACA snoRNAs and tRNAs demonstrated a shift in size distribution between normal and malignant samples (Figure 1d, 1e and Supplementary Figure 3) suggesting cancer-associated alterations in sncRNA processing.

Comparison of the sncRNA composition of the FFPE library with its fresh-frozen counterpart demonstrated that the relative miRNA read-content in FFPE decreased 3.9-fold from 92% to 24% of the total annotated reads. On the contrary, the number of reads mapping to other sncRNA species was strongly elevated *i.e.* sequence read-counts were increased 152-fold for snRNAs, 12.7-fold for H/ACA-box snoRNAs, 5.6-fold for tRNAs, and 2.7-fold for C/D-box snoRNAs (χ^2^ test, p < 0.0001 for all tested groups) (Figure 1c, Supplementary Figure 2 and Supplementary Table 4). The size distribution of read-length in FFPE material was also strongly affected for all examined ncRNA groups except for miRNAs. (Figure 1e, 1f and Supplementary Figure 3). These observations can be explained with the higher level of RNA degradation in FFPE for transcripts longer than miRNA [35, 36].

### Mapping, and annotation of sncRNA-derived RNAs (sncdRNAs)

The majority of miRNA reads in small RNA sequencing data map to the specific location on their pre-miRNA corresponding to the mature miRNA. Similarly, reads mapping to other sncRNAs, originate from specific positions on their precursor rather than being randomly derived and can represent specific, biologically functional, smaller RNA species, *e.g.* sdRNAs or tRNA fragments (tRFs) [37]. Nevertheless, the assignment of RNA-seq sequence-reads to specific sdRNAs or tRFs for quantitation purposes is hampered by the lack of proper annotation. Furthermore, many sncRNAs produce multiple fragments [29, 30] that may overlap each other, which further complicates the exact determination of their origin loci and a subsequent quantitative analysis.

To correctly determine the boundaries of sdRNAs, tRFs and other sncRNA-derived RNAs (sncdRNAs) in our dataset and annotate their specific location on the precursor sequence, we applied the computational algorithm Fragment Location Annotation and Identification Mapper (FlaiMapper) and evaluated its performance in this data set as described [38]. Shortly, FlaiMapper predicted 5′- and 3′-miRNA ends were compared with the 5′- and 3′-end boundaries of corresponding mature miRNAs in MiRBase, v17 [39]. 82% of the detected miRNAs had a correctly determined 5′-end exactly matching miRBase annotation. An additional 11 % had an offset of 1 nt. In agreement with previous observations [39], 3′-ends of mature miRNAs had higher variability and matched miRBase annotations for 45%. From the investigated miRNAs additional 33% had 1 nt offset, and 14%, 2 nt offset (Supplementary Figure 4).

**Figure 2:**
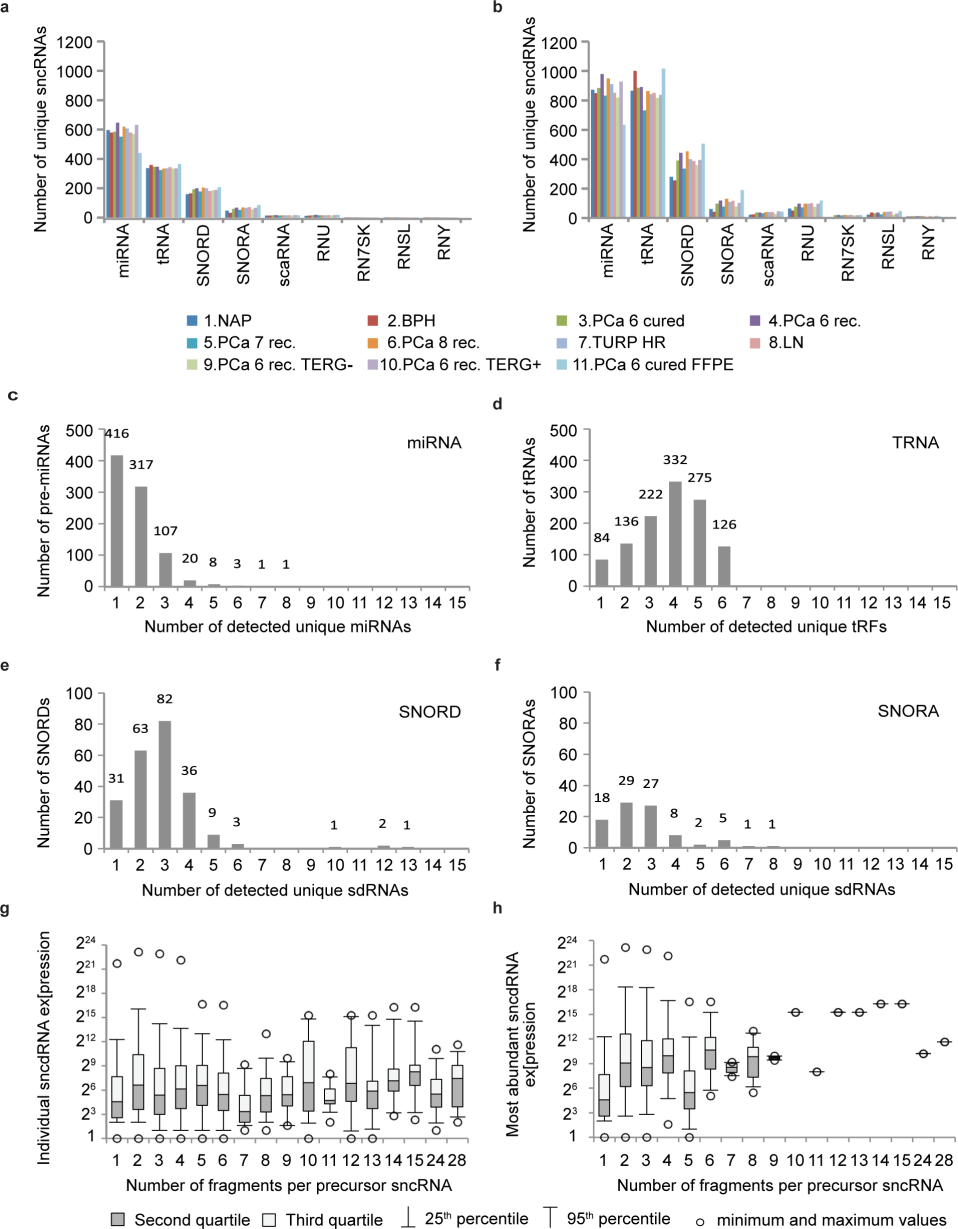
FlaiMapper results (**a**) Total number of detected sncRNA precursors per RNA type and sequencing library. (**b**) Total number of sncdRNAs per precursor type and sequencing library. (**c, d, e, f**) Different types of sncRNAs produce different number of fragments. (**g**) Relation between the number of fragments produced per precursor RNA and the expression levels of individual fragments. (**h**) Relation between the number of fragments produced per precursor RNA and the expression levels of the most abundant fragment per precursor.

Given the high confidence with which FlaiMapper identified 5′- and 3′-end boundaries of *bona fide* miRNAs, we performed annotation of all sncdRNAs in our fresh-frozen libraries. We detected 3927 unique sncdRNAs derived from different precursor classes. From these, 1524 originated from miRNAs, 1175 - from tRNAs, 657 – from C/D-box snoRNAs, 244 – from H/ACA-box snoRNAs, and 327 – from other sncRNA species (Supplementary Table 5 and Supplementary File 2). The total number of detected unique sncdRNAs was higher than the number of detected unique precursor species showing that individual sncRNA precursors produce more than one sncdRNA (Figure 2a, 2b and Supplementary Figure 5). For example, the majority of pre-miRNAs produced one or two miRNAs corresponding to the guide and passenger strand. For C/D box snoRNAs we detected between 1 and 6 sdRNAs originating from the same precursor, with the exception of the unusually long *SNORD3A*, *SNORD3B* and *SNORD3C*, which give rise to 10 to 13 C/D-sdRNAs. Most H/ACA-box snoRNAs produced between 1 and 3 sdRNAs, while for tRNAs we detected between 1 and 6 tRFs per precursor (Figure 2d-2f). Other examined sncRNAs in our libraries produced a varying number of fragments ranging from 3 for the telomerase RNA component to 28 for the small nuclear 7SK RNA (Figure 2c; Supplementary Figure 6 and 7).

We next argued that the expression level of the sncRNA-precursor might positively influence the number of sncdRNAs detected per sncRNA. We examined the distribution of expression values of individual sncdRNAs in relation to the number of sncdRNAs derived per sncRNA and could not observe a strong dependency between the median expression levels of sncdRNAs and the total number of sncdRNAs produced per sncRNA. We obtained similar results when the expression level of the most abundant sncdRNA per precursor was used as a surrogate measure of the expression of the precursor RNA (Figure 2g, 2h). Based on these results, we can conclude that multiple sncdRNAs originating from the same sncRNA can be detected independently of their (low) expression level or the expression level of their precursor. Vice versa, different precursor RNAs can produce only one sncdRNAs with very high abundance. Hence, it can be assumed that the number and quantity of different sncdRNAs do not directly reflect the abundance of their precursor but, like miRNAs, are probably also influenced by additional aspects of cellular metabolism, *e.g.* association with protein complexes and/or turnover rates.

The size of unique sdRNAs ranged between 15 and 29 nt (Supplementary Figure 6). However, when the expression of individual sdRNAs of the same length were accounted, we observed a predominant size of 23 nt for the majority H/ACA-sdRNAs and a binominal size distribution for C/D-sdRNAs with two predominant sizes of 22-23 nt and 28 nt (Supplementary Figure 7), which is in agreement with our previous findings and other reports [3, 22, 23, 40]. C/D-sdRNAs demonstrated a broader size distribution, which however could be a reflection of the broader size range of their precursors.

### sdRNAs are differentially expressed in prostate cancer

**Figure 3:**
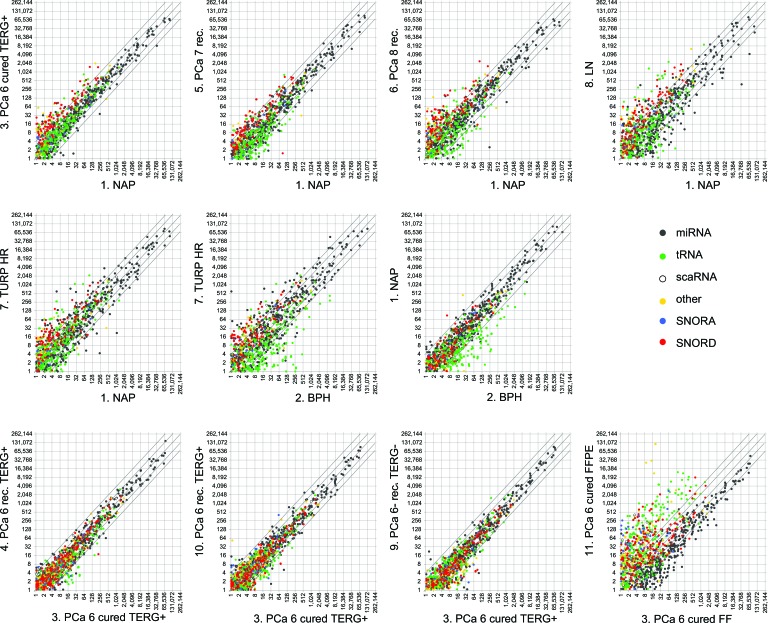
Global expression changes of sncdRNAs in normal and malignant prostate tissue Upper and middle panels present scatterplots comparing the normalized expression values of individual sncdRNA (dots) in each prostate cancer library (PCa) during progressing disease to these in the library prepared from normal adjacent prostate tissue (NAP). The expression of sncdRNAs in the hormone-refractory, transurethral resection of the prostate (TURP HR) library is also compared to the benign prostate hyperplasia (BPH) library since the latest represents the normal counterpart of malignant transurethral resection of the prostate material. Differences in sncdRNA expression between biological replicates of Gleason 6 cancers (PCa 6) as well as comparison of a fresh-frozen library (FF) with its formalin-fixed, paraffin-embedded (FFPE) counterpart derived from the same patients are presented in the lower panels. Each sncdRNA type is presented by a different color. Diagonal lines across each scatterplot represent fold change difference in expression. Middle line, crossing the horizontal and vertical axes at 0, no expression change; lines crossing the vertical and horizontal axes at 2, two-fold expression change; lines crossing the vertical and horizontal axes at 4, four-fold expression change. Cured, no disease relapse after radical prostatectomy; rec., recurrent disease, biochemical or metastatic relapse after surgery; LN, metastatic lymph node sample; TERG+, TMPRSS2-ERG fusion gene event; TERG-, no TMPRSS2-ERG fusion event; Numbers (6, 7 or 8) after PCa indicate the pathological Gleason score of the tumors in the respective group.

Previously, we observed differential expression of sdRNAs between PCa specimens [3]. To examine if such changes are a cancer-specific event we compared the expression of FlaiMapper defined sncdRNAs between normal (NAP and BPH) and malignant tissues of progressing disease (PCa, LN, TURP). We detected between 34 and 202 sncdRNAs with significant differential expression (Table 1, Figure 3, and Supplementary File 3). Approximately one third of the differentially expressed RNAs in each comparison comprised C/D-sdRNAs upregulated in cancer (Figure 3). In contrast, only one sdRNA was differentially expressed between non-malignant samples (NAP and BPH) and only five, between biological replicate samples (PCa, Gleason 6, groups 3, 4, and 10). This suggests that the accumulation of C/D-sdRNAs is primarily driven by malignant transformation.

**Table 1:** Number of differentially expressed sncdRNAs

Comparison	Total number of sncdRNAs with significantly changed expression				Type of sncRNA				
	Corrected p-value^1^(≤ 0.01)	Fold change(≥ ±4)	Expression in cancer(2^nd^ group)		tRF	miRNA	sdRNA		Other
							SNORD	SNORA	
**NAP vs. PCa 6 cured** **(gr1 vs. gr 3)**	**200**	**68**	Up	66	25	9	23	5	4
			Down	2	0	2	0	0	0
**PCa 6 cured vs**.**PCa 6 recurrent** **(gr3 vs. gr4)**	**155**	**14**	Up	0	0	0	0	0	0
			Down	14	10	3	1	0	0
**PCa 6 recurrent vs**.**PCa 6 recurrent** **(gr4 vs. gr10)**	**156**	**16**	Up	8	0	4	1	1	2
			Down	8	0	5	3	0	0
**PCa 6 cured vs**.**PCa 6 recurrent** **(gr3 vs. gr10)**	**141**	**18**	Up	5	0	3	0	1	1
			Down	13	3	7	3	0	0
**NAP vs. PCa 7 recurrent** **(gr1 vs. gr 5)**	**168**	**34**	Up	18	2	3	9	3	1
			Down	16	5	8	1	0	2
**NAP vs. PCa 8 recurrent** **(gr1 vs. gr6)**	**238**	**105**	Up	87	27	15	29	5	11
			Down	18	7	8	1	0	2
**NAP vs. metastatic LN** **(gr1 vs. gr 8)**	**302**	**157**	Up	115	36	28	35	5	11
			Down	42	7	35	0	0	0
**NAP vs. TURP HR** **(gr1 vs. gr 7)**	**254**	**104**	Up	87	24	38	18	1	4
			Down	17	3	11	1	0	2
**BPH vs. TURP HR** **(gr2 vs. gr7)**	**352**	**202**	Up	92	21	42	21	2	6
			Down	110	94	15	0	0	1
**NAP vs. BPH** **(gr1 vs. gr2)**	**250**	**107**	Up	99	93	5	0	0	1
			Down	8	0	5	1	0	2
**FF vs. FFPE** **(gr3 vs. gr11)**	**689**	**540**	Up	462	261	6	80	56	59
			Down	78	2	73	2	1	0

1 Z-test, Bonferroni corrected p-value

To examine the effect of sample storage on fragment abundance we compared the expression of sncdRNAs between the FFPE sample and its fresh-frozen (FF) counterpart. We limited comparison analyses to sncRNAs detected in any of the FF libraries. MiRNAs had decreased expression in FFPE compared with sdRNAs, tRFs and other sncdRNAs (Table 1, Figure 3 and Supplementary Figure 8). Nevertheless, the reduction of miRNA expression in FFPE appears to be the result of a global decrease in miRNA read-counts compared to read-counts of other sncdRNAs (Figure 1c) since the relative expression of miRNAs correlated strongly between both conditions (Pearson ρ = 0.9289) (Supplementary Figure 8). This was not observed for sdRNAs (Pearson ρ = 0.6557 for C/D-sdRNAs and 0.3895 for H/ACA-sdRNAs) or other sncdRNAs, which have longer precursors and may be more susceptible to degradation in FFPE material.

### SdRNAs demonstrate specific global processing patterns in prostate tissue

**Figure 4:**
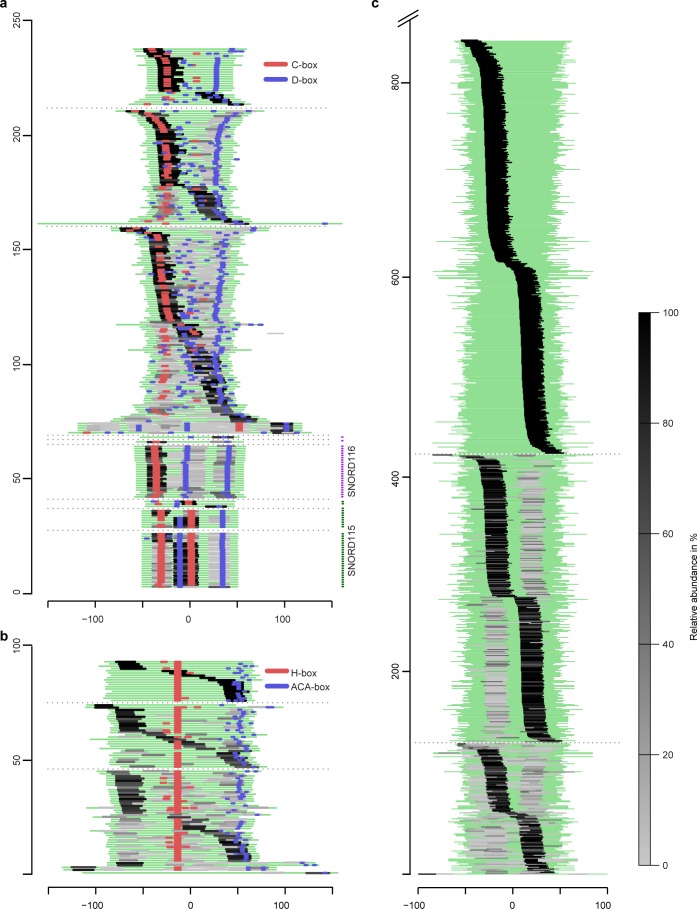
Global processing patterns and relative abundance of sdRNAs and miRNAs expressed in prostate (cancer) tissues (**a**) Full-length C/D-box snoRNAs are aligned relative to the middle nucleotide of each sequence. (**b**) H/ACA-box snoRNAs are aligned based on the position of the H-box. (**c**) Pre-miRNAs are aligned relative to the middle nucleotide of each sequence. A green line represents each full-length sncRNA. Sequences are extended 10 nt at each end to avoid mapping ambiguity caused by incorect annotation. Positions of detected conserved H/ACA-boxes or C/D-boxes are shown in blue and red. Light and dark grey lines indicate the positional origin of sdRNAs, miRNAs and miRNAs*. The color intensity corresponds to the relative abundance of sncdRNAs originating from the same precursor (read-count as a percentage of the total read-count per precursor), *e.g.* if only one sdRNA per snoRNA-precursor is detected it is assigned 100% abundance, if two or more sdRNAs originate from the same snoRNA the sdRNA with the highest read-count is given the darkest color and the sdRNA with the lowest read-count - the lightest. Thin dashed lines separate each panel into three subgroups where sncRNAs producing only one sncdRNA are on top, sncRNAs producing two sncdRNAs are in the middle and those producing there or more sncdRNAs are on the bottom. The highly sequentially conserved, multiple gene-copy C/D-box snoRNAs from the *SNORD116 (HBII-85)* and *SNORD115 (HBII-52)* families are grouped together below other C/D-box snoRNAs. The X-axis indicates the position of sncdRNAs relative to the center of their precursor sequence. The Y-axis depicts the number of full-length sncRNA precursors.

Given the discrete size and specific expression of sdRNAs, we examined detected snoRNAs for the presence of a common processing pattern. To be able to compare with miRNAs, we aligned all snoRNA and pre-miRNA sequences and visualized the position and relative abundance of the corresponding sdRNAs and miRNAs (Figure 4, Supplementary file 4 and 5). The majority of sdRNAs originated from equivalent locations of their precursors. Often, one predominant sdRNA was observed per precursor. The position of these predominant sdRNAs was not dependent on the total number of smaller species detected per precursor sequence, showing a rather uniform fragmentation pattern consistent with the precursor-type. This is in agreement with previously suggested specific snoRNAs processing and accumulation of smaller RNAs observed in cell lines [28, 32].

In our patient samples, predominant H/ACA-sdRNAs originate from either the 5′-arm of the first H/ACA-snoRNA hairpin (38.5%) or the 3′-arm of the second hairpin including the region of the ACA-box (31%) (Figure 4b). C/D-box snoRNA produce twice as many predominant sdRNAs originating from the 5′-terminus that contain a C-box (60.1%) compared to 3′-terminal sdRNAs that contain a D-box (30.1%) (Figure 4a).

Interestingly, individual C/D-sdRNAs with highly similar sequences demonstrate almost identical fragmentation pattern, which is also dependent on the conservation of snoRNA structural features. For example, snoRNAs from the highly conserved, multiple gene-copy *SNORD116* family (*HBII-85*), which have a degenerated C’-box (UGA*G*UGA) produce four sdRNAs where the most abundant one maps to the 5′-region covering the C-box. SnoRNAs from the *SNORD115* (*HBII-52*) family with conserved C’/D’-boxes produce three sdRNAs, with the most abundant ones mapping to the middle-region and covering the entire K-loop including the C’/D’-box. In contrast, the larger snoRNAs from the *SNORD3* family, which lack a conserved C-box, produce between 10 and 13 overlapping sdRNAs with the most predominant mapping to the 3′-end.

*SNORD115* and *SNORD116* sdRNAs differ in size and position from the previously reported highly abundant psnoRNAs processed from the orthologous *MBII-52* and *MBII-85* detected by RNase protection assays [29, 30, 41]. This discrepancy could be explained by the implicit methodology differences between sncRNA sequencing and RNase protection assays. However, these differences could be also caused by tissue-specific sdRNA accumulation as previously described for sdRNAs originating from *SNORD88C* (*HBII-180C*) [32] or by the dependence of processing mechanisms on the structural conservation of C/D-box snoRNAs. Of note, *SNORD115*, *SNORD116*, or *SNORD88C*-originating sdRNAs were detected at low abundance in our samples.

### Processing and expression of sdRNAs originating from GAS5 encoded C/D-box snoRNAs is related to the conservation of structural C’/D’-boxes

**Figure 5:**
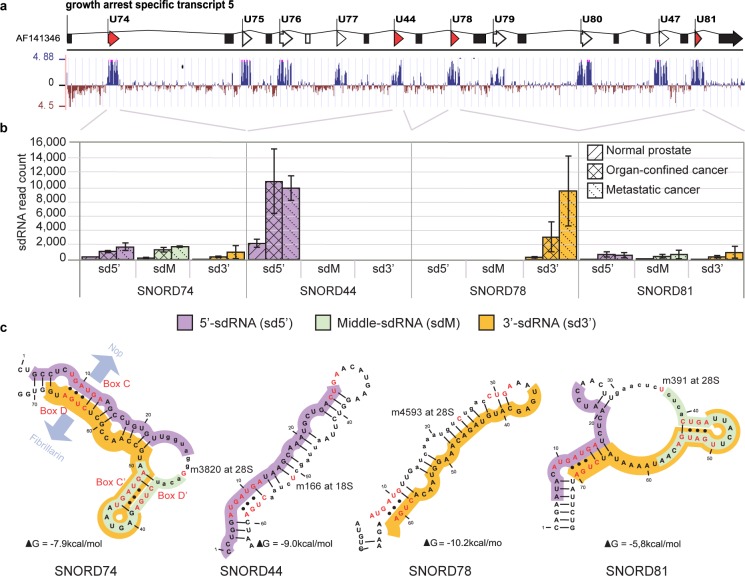
Genomic organization, conservation, secondary folding, fragmentation pattern and expression of *SNORD44, SNORD74, SNORD78*, and *SNORD81* (**a**) *SNORD44*, *SNORD74*, *SNORD78*, and *SNORD81* are transcribed simultaneously from the conserved intronic regions of the protein-non-coding *GAS5* gene. Spliced exons (boxes); snoRNA loci (arrows). (**b**) sdRNAs from *SNORD44*, *SNORD74*, *SNORD78*, and *SNORD81* are up-regulated in PCa. Expression is comparable to PCa-relevant microRNA (not shown). SNORD74 and SNORD81 produce equally expressed 5′- (sd5′), middle- (sdM), and 3′-sdRNAs (sd3′) with overlapping sdM and sd3′. SdRNAs originating from the middle regions extend towards the antisense box and their 5′-ends map exactly adjacent to the nucleotide complementary to the targeted ribosomal residue. (**c**) *SNORD44* and *SNORD78* produce predominantly one sdRNA either from the 5′- or the 3′-arm of the snoRNA. The position of core snoRNP-proteins NOP58/56 and FIBRILLARIN (indicated at *SNORD74*) is dependent on the kink-turns formed by non-complementary base-pairing (dots) of the conserved external sequence boxes C and D and/or the internal boxes C’/D’. The rRNA-complementary antisense-box (lower case) is exposed and contains the nucleotide targeted for modification (red), positioned exactly 5 nt upstream of the D or D’ box.

We investigated whether the fragmentation pattern of other C/D-box snoRNA is also dependent on structural feature conservation. For this we analyzed the positional origin of a highly abundant sdRNA produced from the 3′-end of *SNORD78* [3] and other sdRNAs from the same locus. *SNORD78* is intronically encoded by the Growth Arrest Specific 5 gene (*GAS5*) together with 9 other C/D-box snoRNAs [42]. All 10 SNORDs are presumably simultaneously transcribed as a *GAS5* precursor-transcript, which undergoes intron removal and posttranscriptional processing. We could detect sdRNAs from all 10 *GAS5*-encoded snoRNAs. However, only four (*SNORD44*, *SNORD78*, *SNORD74* and *SNORD81*) snoRNAs produced abundant sdRNAs (Figure 5 and Supplementary Figure 9).

Interestingly, *SNORD74* and *SNORD81* produced three abundant sdRNAs with similar, relatively low expression levels that mapped to the 5′-, 3′-, and middle region of the snoRNAs. The 3′- and middle sdRNAs overlapped each other and covered the K-loop and the conserved canonical C’/D’-box (Figure 5). In contrast, *SNORD78* and *SNORD44*, which lack the canonical C’/D’-box, produced predominantly one 28 nt long sdRNA each, mapping to the 3′-arm for *SNORD78* (*sd78-3′*) or the 5′-arm of *SNORD44* (*sd44-5′*). *sd78-3′* and *sd44-5′* were strongly upregulated in samples prepared from malignant tissue compared to normal or benign, while middle- and opposite arm-derived sdRNAs were present only at very low read-counts in all libraries (Supplementary Figure 9a and 10).

### SNORD78 and *sd78-3*’ expression is associated with metastatic PCa

**Figure 6:**
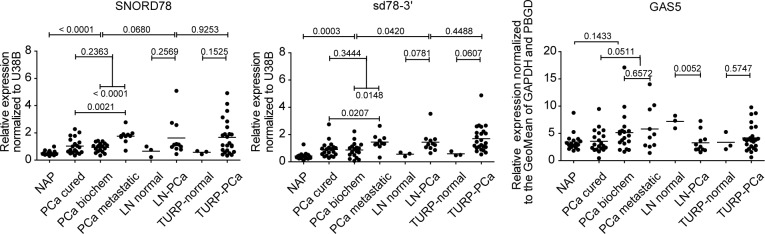
Q-PCR validation of snoRNA and sdRNA expression in an independent cohort of patient samples NAP, normal adjacent prostate (n=17); PCa-cured, radical prostatectomy sample, no disease relapse after radical prostatectomy (n=20); PCa-biochem, radical prostatectomy sample, patients manifested biochemical disease relapse after surgery (n=18); PCa-met, radical prostatectomy sample, metastatic disease progression after surgery (n=10); LN-normal, normal lymph node (n=3); LN-PCa, metastatic lymph node (n=11); TURP-normal, transurethral resection of the prostate sample that does not contain cancer cells (n=3). PCa-TURP, transurethral resection of the prostate sample that contains cancer cells (n=24). Horizontal line marks the mean of each group. Patient number in each group is indicated in brackets. P-values from unpaired two-tailed t-tests (alpha level 0.05) are indicated above each comparison.

To validate our sequencing data we tested the expression of *SNORD44*, *SNORD78*, *SNORD74*, *SNORD81*, and their derivate sdRNAs, in an independent patient cohort of 106 fresh-frozen clinical samples by quantitative real-time PCR (Q-PCR). To evaluate whether increased sdRNA expression is a result of a general activation of the *GAS5* locus, we also measured the expression of the spliced *GAS5* transcript (Figure 6 and Supplementary Figure 9b). All tested snoRNAs and sdRNAs were upregulated in organ-confined PCa compared to normal adjacent controls. This was not related to an elevation of the spliced GAS5 transcript, which did not demonstrate pronounced expression changes between NAP and PCa. Interestingly, overlapping sdRNAs originating from the same snoRNA as well as full-length snoRNAs were simultaneously detectable by Q-PCR suggesting the existence of multiple conformational states of these snoRNAs.

Sd78-3′, SNORD78 and GAS5 expression was also detectable in different normal basal prostate epithelium cell lines (PNT2C2, RWPE) prostate cancer cell lines (PC346C, LAPC4, VCAP, LNCAP, 22RV1, PC3, and DU145N) as well as in hepatocellular carcinoma (HEP3B) and colon adenocarcinoma (COLO205) cells demonstrating that SNORD78 processing to sd78-3′ is not restricted to prostate tissue or cells. Similarly to patient data, the expression levels of sd78-3′ and SNORD78 were not correlated to the expression of the GAS5 host gene (Supplementary Figure 11).

Consistent with our previous results [3] *sd78-3′* was upregulated in the LN library generated in this study, suggesting association of this sdRNA with aggressive disease. Therefore, in the validation cohort we stratified patients with organ-confined disease at the time of radical prostatectomy into three groups: cured after radical prostatectomy, biochemical disease reoccurrence, and progression to metastatic disease after surgery. Strikingly, the expression of *sd78-3′* and its precursor *SNORD78* in the third group was significantly higher already at the time of surgery, suggesting an early involvement in PCa progression and possible prognostic marker potential for these sncRNAs.

## DISCUSSION

SncRNAs and in particular miRNAs emerged as novel modulators of gene expression and regulators of fundamental cellular processes often disturbed in cancer. At the same time, long-known “housekeeping” RNAs such as snoRNAs appeared to have tissue-specific expression altered in solid tumors and hematological malignances [8, 36, 43]. Furthermore, several studies discussed above demonstrate that similarly to miRNA, snoRNAs carry diagnostic and/or prognostic biomarker potential in different cancer types [6-10, 14, 15].

Improved detection and screening over the last decade led to large increase in prostate cancer detection. However, the majority of presently diagnosed patients carry clinically insignificant tumors, which would never progress to a life threatening disease. Without the presence of better prognostic markers, many patients undergo unnecessary invasive surgical treatment.

Prompted by our previous findings on elevated levels of snoRNA fragments in metastatic PCa [3] and by accumulating evidence from sequencing data that demonstrates processing of snoRNA to stable smaller sdRNAs [22, 32, 37] we combined RNA sequencing of human prostate (cancer) tissue with tailored computational analysis. This resulted in a methodologically quantitated catalog of 3927 sncdRNAs originating from 1637 unique sncRNAs and allowed us to follow for changes in their expression during malignant transformation and cancer progression.

To investigate possible effects of sample storage conditions we compared the sncRNA transcriptome of fresh-frozen tissue with its FFPE-stored counterpart. We saw large changes in the accumulation of sncdRNAs, particularly sdRNAs and tRFs, when we compared fresh-frozen with FFPE material. This was not the case for miRNAs were we observed only relative down-regulation most possibly caused by the additional buildup of degradation products of mRNAs and long ncRNAs due to sample preparation and storage [35]. In addition, while miRNA read-length in FFPE tissue remained unchanged, reads from other sncRNAs had changed length distribution indicating that FFPE-preserved tissue is less suitable for the analysis of sncRNAs other than miRNA.

Previously we detected differential expression of sdRNAs between organ-confined PCa and lymph node metastases [3]. The expression analysis presented here indicates that the major accumulation of sdRNAs is associated with malignant transformation and can be described by an increased global production and/or accumulation of sdRNAs already in the early cancer stages but it is not directly associated with the expression levels of precursor snoRNAs. Biological replicate analysis among three libraries (PCa, Gleason score 6) confirms the reproducibility of sequencing experiments on fresh-frozen tissue as less than 20 sncRNAs show significantly changed expression levels. We did not observe a direct association between the number of sncdRNAs arising from one precursor and its quantity suggesting that sncdRNA accumulation is not the direct result of increased sncRNA turnover in malignant cells. Q-PCR analysis confirmed the expression changes detected by sequencing and also identified the simultaneous existence of full-length snoRNAs and their derivate sdRNAs from the GAS5 locus. The high levels of SNORD78 and sd78-3′ in a subset of patients, which progressed to metastatic disease, identify these two sncRNAs as possible novel prognostic biomarkers for the further stratification of PCa patients at high risk of developing aggressive disease.

It has been shown that the majority of C/D-sdRNAs are derived from the termini of their precursor and may remain attached to the core snoRNP shielded from further degradation [28]. A large part of the sdRNAs detected in our libraries is also terminally derived. Nevertheless, the processing patterns of snoRNAs that we observe, and the accumulation of specific sdRNAs appear to be dependent on the conservation of structural snoRNA features and do not always correspond to snoRNA termini protected by the snoRNP. Furthermore, the overlap and discrete origin-position of multiple sdRNAs produced from the same precursor exemplified by sdRNAs produced from *SNORD44*, *SNORD78*, *SNORD74* and *SNORD81* suggest rather specific nucleolytic cleavage that requires different conformational states for C/D-box snoRNAs [44] possibly assisted by structural interaction with the core snoRNPs or yet unidentified proteins. Of note, the highly abundant *sd78-3′* is derived from the opposite part of *SNORD78* and does not overlap with the previously reported snoRNP footprint of *SNORD78* observed by Kishore *et al* [28]. It has been proposed that the specificity of sdRNA processing patterns detected in human cell lines is conserved between different cell types while the accumulation of individual sdRNAs is cell type specific implying the existence of dedicated processing mechanisms [22, 23, 32, 45].

It remains to be established how sdRNAs and other sncdRNAs are produced in the cell and to what extend this process is deregulated in cancer. The miRNA processing RNaseIII, DICER was suggested in the biogenesis of H/ACA-box-originating sdRNAs that have an apparent size of 20-24 nt. However, C/D box-sdRNAs identified by us and others [22, 23] have a bimodal size distribution which deviates from that of Dicer products, suggesting the involvement of other nuclease(s) in the generation of sdRNAs. Another protein from the miRNA biogenesis pathway that could be involved in the generation of SNORD-sdRNAs is AGO2. It has been shown that AGO2 is responsible for the maturation of pre-miRNA-451 which is too short to undergo Dicer processing. The AGO2 cleaved miR-451 product is a fragment of 30 nt that is processed further to the mature 23 nt long miR-451 by unknown exonucleases [46]. Nonetheless, AGO2-derived mature miR-451 is predominantly uridilated at is 3′-end, while most of the C/D box-sdRNAs in our libraries are not. Furthermore, recent analyses of AGO2 PAR-CLIP libraries demonstrate that despite their cellular abundance, C/D box snoRNAs-originating sdRNAs are not efficiently incorporated in AGO2 [28].

The small transcriptome is a mix of turnover products and functional entitles, where a proportion of the cellular sdRNA pool most probably represents stable degradation products shielded by effector proteins. Nevertheless, the mechanisms of sdRNA generation and their putative functional role in normal and malignant cells should be investigated further alongside with their biomarker potential in prostate and other cancers.

## MATERIALS AND METHODS

### Patient samples and cell lines

Snap-frozen, liquid nitrogen stored and FFPE clinical samples (Supplementary Table 1) were from the tissue bank of the Erasmus University Medical Center, Rotterdam, The Netherlands and from Tampere University Hospital (TAUH), Tampere, Finland. Collection and use of patient material was performed according to the national legislations concerning ethical requirements and approved by the Erasmus MC Medical Ethics Committee, Medical Research Involving Human Subjects Act (MEC-2004-261), and the Ethical Committee of the Tampere University Hospital.

Prostate and lymph node tissues were from radical prostatectomy. BPH samples were obtained from cystoprostatectomies and found not to contain any prostate cancer cells. PCa-TURP samples were collected by transurethral resection of the prostate. Histological evaluation of analyzed material was described previously [3].

### RNA isolation

Total RNA from frozen tissue was isolated using RNABee reagent (Campro Scientific, GmbH, Berlin, Germany) according to manufacturer's protocols. Total RNA isolation from FFPE material was described previously [37].

### Sequencing

Total RNA sample pools of four individual patient samples each, were outsourced (BGI, Shenzhen, China) for sequencing. Library preparations were performed according to the “Small RNA Sample Preparation Guide, Part #1004239”, (Illumina Inc., http://www.illumina.com). Shortly, total RNA pools were separated on 15% Tris/Borate/EDTA urea polyacrylamide electrophoresis gel, and the sncRNA fraction in the size range of 15 to 35 nt was extracted and purified. After 5′- and 3′-adapter ligation, cDNA was generated by reverse transcription with SuperScript II Reverse Transcriptase (Invitrogen, Carlsbad, CA, USA) followed by 15 cycles of PCR by Phusion DNA Polymerase (Finnzymes Oy, Espoo, Finland).

### Small non-coding RNA database (sncRNAdb)

Official small non-coding RNA nomenclature lists and NCBI RefSeq identifier numbers for microRNA precursors (pre-miRNAs), small nucleolar RNAs (snoRNAs), small cytoplasmic RNA (scRNAs), small nuclear RNA (snRNAs), and small miscellaneous RNAs (miscRNAs) were retrieved from the HUGO Gene Nomenclature Committee (HGNC) (http://www.genenames.org) [47]. Genome locations corresponding to the RefSeq entries were further extended with 10 nt at the 5′- and 3′-end to ensure correct mapping of reads derived from ambiguously annotated ncRNAs and mapped against the Human Genome Browser – hg19 assembly at the University of California Santa Cruz (UCSC) (http://genome.ucsc.edu).[48] UCSC Genome Browser uses miRBase 15; therefore all miRNAs entries were manually curated to match miRBase 17.

Since HGNC does not provide RefSeq identifiers for tRNAs, tRNA data was retrieved from the UCSC dedicated Genomic tRNA Database (http://gtrnadb.ucsc.edu/) [49]. The number of mapped reads was positively influenced by the addition of “CCA” triplet to the 3′-end of genomic tRNA sequences and intron removal. Therefore, tRNA entries represent the mature tRNA form and are not extended. Sequences, genomic loci and database identifiers of all ncRNAdb entries are given in Supplementary File 1).

### Computational analysis of sequencing data

Initial mapping of sequencing reads to sncRNAdb was done in CLC-Bio Genomics Workbench (v. 4.9) following the “Small RNA Analysis” workflow. Read-summarizing and adapter-removal parameters from the “Extract and Count” tool were applied: Minimum sampling count was 4; Minimum and maximum number of nucleotides in reads was 15 and 35 nt, respectively; no 3′- or 5′- terminal nucleotide removal was performed. Each read was screened with “no fixed adapter length” for the (partial) presence of Illumina small RNA adapter: CAAGCAGAAGACGGCATACGA on the minus strand with alignment mismatches and gaps allowance at a mismatch cost of 3, and a gap cost of 5, minimum score: ns, minimum score end: 3. If adapter was not found reads were discarded from further analysis. Filtered sequence reads were mapped to sncRNAdb with a maximum of 2 mismatches allowed using the “Annotate and Merge” tool.

### Location, annotation and quantitation of sncdRNAs

Annotation of sncdRNAs was done using FlaiMapper as described [38]. Only sequence reads from libraries derived from fresh-frozen material were used as an input for the calculation of 5′- and 3′-ends of sncdRNAs. Quantitation and expression analysis of sncdRNAs was performed in a second round of mapping to FlaiMapper annotated sncRNAdb using ‘Small RNA Analysis’ workflow in CLC-Bio Genomics Workbench (v.4.9). “Expression values” that equal the sum of all reads mapping to a FlaiMapper annotated sncdRNA were used. Expression data was normalized with the “Reads per Million” algorithm. Differentially expressed sncdRNAs were detected using Kal's Z-test on proportions [50] with two-sided p-value, followed by Bonferroni correction with a corrected p-value cut-off of 0.01.

### Quantitative real time PCR (qPCR)

snoRNA and sdRNA expression levels were evaluated by qPCR using miRCURY LNA™ Universal RT microRNA PCR, Polyadenylation and cDNA synthesis and SYBR Green kits (Exiqon, Copenhagen, Denmark) and custom LNA™ primers according to the manufacturer's instructions. Custom LNA primers for qPCR analysis of snoRNAs and sdRNAs were designed by Exoqon A/S, Copenhagen, Denmark. Target sequences used for primer design as well as design IDs are listed in Supplementary methods table M1. SNORD38B expression was measured with Reference gene primer set 20391 (Exiquon, Vedbaek, Denmark) and used to normalize raw Ct values by the delta delta Ct Method.

GAS5 expression was assessed by the Promega Reverse Transcription System (Promega Benelux, The Netherlands) and SybrGreen qPCR System (Roche, The Netherlands) according to manufacturer protocols. Primers used were GAS5 FW: CAAGGACTCAGAATTCATGAT and GAS5 REV: AGTGGTCTTTGTAGACTGCC. Raw expression values were normalized against the geometrical mean of GAPDH and PBGD by the delta delta Ct Method.

### Statistical analysis

Significance of sncRNA composition and read-numbers were assessed with chi-square test for independence without Yates' correction. Two-sided p-values were calculated at alpha level of 0.05. Differences between groups in qPCR experiments were tested with unpaired two-tailed t-test at alpha level 0.05. Pearson correlation coefficients were assessed at an alpha level of 0.05 using GraphPad Prism 5.

## SUPPLEEMENTARY MATERIAL TABLES AND FIGURES












