# Supplementary material for: C/D-box snoRNA-derived RNA production is associated with malignant transformation and metastatic progression in prostate cancer
Source: Oncotarget. 2015 May 19;6(19):17430–44. doi: 10.18632/oncotarget.4172 (PMC4627319; doi:10.18632/oncotarget.4172)

C/D-box snoRNA-derived RNA production is associated with malignant transformation and metastatic progression in prostate cancer

Supplementary Material

Supplementary methods table M1. Custom LNA primers for the quantitative real time PCR analysis of snoRNAs and sdRNAs were designed by Exiqon A/S, Copenhagen, Denmark using target sequences listed in Supplementary table M1. Forward and reverse primer sequence positions are marked in color. M' refers to sdRNA derived from the middle of the full SNORD sequence.

ASSAY ID	RNA name	TARGET SEQUENCE 5′ -> 3′
443822	SNORD78 Full length	GTGTAATGATGTTGATCAAATGTCTGACCTGAAATGAGCATGTAGACAAAGGTAACACTGAAGAA
443827	sd78-3'	TGAGCATGTAGACAAAGGTAACACTG
443830	SNORD44 Full length	CCTGGATGATGATAAGCAAATGCTGACTGAACATGAAGGTCTTAATTAGCTCTAACTGACTAA
443836	sd44-5′	CCTGGATGATGATAAGCAAATGCTGACT
443839	SNORD74 Full length	CTGCCTCTGATGAAGCCTGTGTTGGTAGGGACATCTGAGAGTAATGATGAATGCCAACCGCTCTGATGGTGG
443845	sd74-5′	GCCTCTGATGAAGCCTGTGTTGGTA
443848	sd74-M′	ACATCTGAGAGTAATGATGAA
443852	sd74-3'	AGTAATGATGAATGCCAACCGCTCTGA
443855	SNORD81 Full length	CAGAATACATGATGATCTCAATCCAACTTGAACTCTCTCACTGATTACTTGATGACAATAAAATATCTGATATTCTG
443861	sd81-5′	ATACATGATGATCTCAATCC
443864	sd81-M′	CTCACTGATTACTTGATGACAA
443867	sd81-3′	TTACTTGATGACAATAAAATATCTGA

Supplementary table 1. Patient cohort used for the generation of sncRNA libraries[1].

Library	Gleason score	Genomic alterations (TMPRSS2-ERG; ETV1)	Epithelium %	Cancer %	PCa recurrence after radical prostatectomy	Died
**Group 1. NAP**						
Patient 1	n.a.	n.a.	70	0	n.a.	n.a.
Patient 2	n.a.	n.a.	80	0	n.a.	n.a.
Patient 3	n.a.	n.a.	70	0	n.a.	n.a.
Patient 4	n.a.	n.a.	70	0	n.a.	n.a.
**Group 2. BPH**						
Patient 5	n.a.	n.a.	>70%	0	n.a.	n.a.
Patient 6	n.a.	n.a.	>70%	0	n.a.	n.a.
Patient 7	n.a.	n.a.	>70%	0	n.a.	n.a.
Patient 8	n.a.	n.a.	>70%	0	n.a.	n.a.
**Group 3. PCa 6 cured**						
Patient 9	3+3	TMPRSS2-ERG	90	80	No	No
Patient 10	3+3	TMPRSS2-ERG	80	75	No	No
Patient 11	3+3	TMPRSS2-ERG	80	90	No	No
Patient 12	3+3	TMPRSS2-ERG	80	70	No	No
**Group 4. PCa 6 recurrent**						
Patient 13	3+3	TMPRSS2-ERG	80	90	M	Yes
Patient 14	3+3	TMPRSS2-ERG	80	90	PSA progression	-
Patient 15	3+3	TMPRSS2-ERG	70	80	PSA progression	-
Patient 16	3+3	TMPRSS2-ERG	80	85	PSA progression	-
**Group 5. PCa 7 recurrent**						
Patient 17	4+3	TMPRSS2-ERG	80	90	M	Yes
Patient 18	4+3	ETV1 fusion	90	100	M	Yes
Patient 19	4+3	TMPRSS2-ERG	70	100	PSA progression	
Patient 20	4+3	no	70	80	PSA progression	
**Group 6. PCa 8 recurrent**						
Patient 21	4+4	ETV1 overexpression	80	100	M	Yes
Patient 22	4+4	TMPRSS2-ERG	90	90	M	Yes
Patient 23	4+5	no	70	100	M	Yes
**Group 7. PCa TURP hormone refractory disease**						
Patient 24	5+4	no	90	100		HR
Patient 25	3+4	TMPRSS2-ERG	70	90	M	Yes
Patient 26	3+5	no	95	100		HR
Patient 27	5+3	ETV1 fusion	95	100		HR
**Group 8. LN-PCa**						
Patient 28	4+4	TMPRSS2-ERG	n.a.	100	N	Yes
Patient 29	4+5	TMPRSS2-ERG	n.a.	100	N	Yes
Patient 30	4+4	TMPRSS2-ERG	n.a.	100	N	Yes
Patient 31	4+4	ETV1 fusion	n.a.	100	N	yes
**Group 9. PCa 6 recurrent no chromosomal alterations**						
Patient 32	3+3	no	80	90	M	No
Patient 33	3+3	no	80	90	M	Yes
Patient 34	3+3	no	70	70	M	No
Patient 35	3+3	no	90	80	PSA progression	Yes
**Group 10. PCa 6 TMPRSS2-ERG fusion**						
Patient 36	3+3	TMPRSS2-ERG	90	80	M	Yes
Patient 37	3+3	TMPRSS2-ERG	90	90	PSA progression	No
Patient 38	3+3	TMPRSS2-ERG	70	80	No	Yes
Patient 39	3+3	TMPRSS2-ERG	80	90	No	No
**Group 11. FFPE**						
Patient 9	3+3	TMPRSS2-ERG	90	80	No	No
Patient 10	3+3	TMPRSS2-ERG	80	75	No	No
Patient 11	3+3	TMPRSS2-ERG	80	90	No	No
Patient 12	3+3	TMPRSS2-ERG	80	70	No	No

[1] NAP, normal adjacent prostate sample; BPH, benign prostate hyperplasia sample; PCa, radical prostatectomy sample; cured, no disease relapse after radical prostatectomy; recurrent, biochemical or metastatic disease relapse after surgery; LN-PCa, metastatic lymph node sample; PCa-TURP, transurethral resection of the prostate sample; TMPRSS2-ERG, fusion gene event; FFPE, formalin-fixed, paraffin-embedded sample (replicate of group3) n.a, not applicable; M, metastatic occurrence; HR, hormone refractory disease.

Supplementary table 2

	Group 1 NAP	Group 2 BPH	Group 3 PCa 6 cured	Group 4 PCa 6 recurrent	Group 5 PCa 7 recurrent	Group 6 PCa 8 recurrent	Group 7 PCa TURP	Group 8 LN met	Group 9 PCa 6 TERG-	Group 10 PCa 6 TERG+	Group 11 FFPE
Total read number	14,162,933	14,516,262	14,598,363	14,876,075	13,468,284	15,114,031	14,827,046	14,298,445	14,597,918	14,309,106	15,393,670
Removed reads	811,765	868,054	885,824	1,488,564	909,675	1,041,211	1,034,018	999,260	1,091,428	1,658,413	3,545,478
Extracted reads	13,351,168 (94 %)	13,648,208 (94 %)	13,712,539 (94 %)	13,387,511 (90 %)	12,558,609 (93 %)	14,072,820 (93 %)	13,793,028 (93 %)	13,299,185 (93 %)	13,506,490 (93 %)	12,650,693 (88 %)	11,848,192 (77 %)
Annotated reads	11,888,480 (84 %)	12,044,365 (83 %)	11,082,648 (76 %)	10,859,490 (73 %)	11,213,771 (83 %)	11,994,227 (79 %)	11,650,557 (79 %)	10,758,631 (75 %)	11,496,537 (79 %)	9,971,092 (70 %)	8,010,752 (52 %)
Unique reads	551,939	584,853	729,486	598,249	548,038	788,488	762,000	778,904	595,716	631,370	822,911
Annotated ncRNA from ncRNAdb	902	890	936	1023	881	994	977	940	911	990	851

Supplementary table 3. Number of small ncRNA-species detected in each library

Count of precursors per RNA type	Total Count ncRNA type	Group 1 NAP	Group 2 BPH	Group 3 PCa 6 cured	Group 4 PCa 6 recurrent	Group 5 PCa 7 recurrent	Group 6 PCa 8 recurrent	Group 7 PCa TURP	Group 8 LN met	Group 9 PCa 6 TERG-	Group 10 PCa 6 TERG+	Group 11 FFPE	Average Count ncRNA type
MIR	**873**	597	580	585	648	551	620	607	580	569	632	441	**597**
RN7SK	**1**	1	1	1	1	1	1	1	1	1	1	1	**1**
RN7SL	**2**	2	2	2	2	2	2	2	2	2	2	2	**2**
RNU	**23**	13	15	16	19	16	17	17	18	15	18	19	**16**
RNY	**4**	3	3	3	3	3	3	3	3	3	3	3	**3**
RPPH1	**1**	1	1	1	1	1	1	1	1	1	1	1	**1**
SCARNA	**19**	14	15	17	18	15	17	17	17	15	18	17	**16**
SNAR	**4**	0	0	2	1	0	3	1	3	2	1	0	**1**
SNORA	**91**	48	34	59	69	53	70	67	71	56	67	86	**59**
SNORD	**228**	161	166	193	201	178	205	200	181	187	189	208	**186**
TERC	**1**	1	1	1	1	1	1	1	1	1	1	1	**1**
TRNA	**385**	337	358	346	347	324	334	336	345	334	336	366	**340**
VTRNA	**5**	5	5	5	5	5	5	5	5	5	5	5	**5**
**Grand Total**	**1637**	**1183**	**1181**	**1231**	**1316**	**1150**	**1279**	**1258**	**1228**	**1191**	**1274**	**1150**	**1229**

Supplementary table 4. Total number unique mapped reads per type of small ncRNA precursor and sample library

ncRNA	Group 1 NAP	Group 2 BPH	Group 3 PCa 6 cured	Group 4 PCa 6 recurrent	Group 5 PCa 7 recurrent	Group 6 PCa 8 recurrent	Group 7 PCa TURP	Group 8 LN met	Group 9 PCa 6 TERG-	Group 10 PCa 6 TERG+	Group 11 FFPE
pre-miRNA	11,367,682	10,777,063	10,174,066	10,023,933	10,722,355	11,110,642	10,547,062	9,202,300	10,839,999	9,278,102	1,907,837
	(95.6%)	(89.5%)	(91.8 %)	(92.3%)	(95.6%)	(92.6%)	(90.5%)	(85.5%)	(94.3%)	(93.1%)	(23.8%)
tRNA	229,457	894,493	374,392	352,770	212,392	346,797	337,292	602,838	332,218	292,345	1,519,993
	(1.93%)	(7.43%)	(3.38%)	(3.25%)	(1.89%)	(2.89%)	(2.90%)	(5.60%)	(2.89%)	(2.93%)	(18.97%)
Mt-tRNA	40,160	99,840	104,961	75,886	49,921	108,902	197,520	63,653	37,734	97,275	63,852
	(0.34%)	(0.83%)	(0.95%)	(0.70%)	(0.45%)	(0.91%)	(1.70%)	(0.59%)	(0.33%)	(0.98%)	(0.80%)
rRNA	148,179	171,301	172,188	138,057	66,525	152,725	365,820	437,935	102,076	93,744	2,240,371
	(1.25%)	(1.42%)	(1.55%)	(1.27%)	(0.59%)	(1.27%)	(3.14%)	(4.07%)	(0.89%)	(0.94%)	(27.97%)
SNORA	2,127	2,219	7,522	6,955	4,814	12,603	5,440	7,566	4,088	10,236	68,971
	(0.02%)	(0.02%)	(0.07%)	(0.06%)	(0.04%)	(0.11%)	(0.05%)	(0.07%)	(0.04%)	(0.10%)	(0.86%)
SNORD	43,005	41,957	158,927	169,165	103,279	171,873	117,390	199,045	121,528	123,460	308,121
	(0.36%)	(0.35%)	(1.43%)	(1.56%)	(0.92%)	(1.43%)	(1.01%)	(1.85%)	(1.06%)	(1.24%)	(3.85%)
scaRNA	13,624	14,208	12,622	18,053	14,469	19,856	25,066	22,528	12,890	12,698	10,720
	(0.11%)	(0.12%)	(0.11%)	(0.17%)	(0.13%)	(0.17%)	(0.22%)	(0.21%)	(0.11%)	(0.13%)	(0.13%)
scRNA	26,293	37,140	53,364	46,948	30,222	42,837	37,215	59,128	33,493	38,107	37,952
	(0.22%)	(0.31%)	(0.48%)	(0.43%)	(0.27%)	(0.36%)	(0.32%)	(0.55%)	(0.29%)	(0.38%)	0.47%)
snRNA	15,849	4,953	16,729	21,181	7,945	23,844	14,887	151,045	7,876	19,115	1,839,129
	(0.13%)	(0.04%)	(0.15%)	(0.20%)	(0.07%)	(0.20%)	(0.13%)	(1.40%)	(0.07%)	(0.19%)	(22.96%)
miscRNA	2,080	1,172	7,854	6,505	1,833	4,076	2,840	12,542	4,577	5,489	13,605
	(0.02%)	(0.01%)	(0.07%)	(0.06%)	(0.02%)	(0.03%)	(0.02%)	(0.12%)	(0.04%)	(0.06%)	(0.17%)
TERC	24	19	23	37	16	72	25	51	58	521	201
	(0.00%)	(0.00%)	(0.00%)	(0.00%)	(0.00%)	(0.00%)	(0.00%)	(0.00%)	(0.00%)	(0.01%)	(0.00%)
**Total**	**11,888,480**	**12,044,365**	**11,082,648**	**10,859,490**	**11,213,771**	**11,994,227**	**11,650,557**	**10,758,631**	**11,496,537**	**9,971,092**	**8,010,752**

Supplementary table 5. Number of detected sncdRNAs (e.g. sdRNAs, tRFs and other fragments) per type of RNA precursor

Type of precursor small ncRNA	Total Count unique smaller RNAs	Group 1 NAP	Group 2 BPH	Group 3 PCa 6 cured	Group 4 PCa 6 recurrent	Group 5 PCa 7 recurrent	Group 6 PCa 8 recurrent	Group 7 PCa TURP	Group 8 LN met	Group 9 PCa 6 TERG-	Group 10 PCa 6 TERG+	Group 11 FFPE	Average Count unique smaller RNAs
MIR	**1524**	872	850	885	980	832	948	911	852	818	928	634	**865**
TRNA	**1175**	866	1000	886	891	732	863	843	852	816	837	1017	**873**
SNORD	**657**	280	255	391	443	336	453	400	387	359	394	506	**382**
SNORA	**244**	61	41	97	119	77	131	109	116	79	103	189	**102**
SCARNA	**74**	23	25	36	36	32	40	40	40	26	46	43	**35**
RNU	**129**	64	50	76	98	71	97	97	100	74	98	119	**86**
RN7SK	**27**	3	6	19	21	17	21	19	21	17	19	21	**17**
RN7SL	**48**	22	37	32	36	25	40	42	43	23	29	47	**34**
RNY	**15**	10	10	12	12	12	12	9	12	10	14	11	**11**
RPPH1	**11**	7	3	1	7	2	7	9	11	4	5	9	**6**
SNAR	**4**	0	0	2	1	0	3	1	3	2	1		**1**
TERC	**3**	2	1	1	2	1	2	1	1	1	2	2	**1**
VTRNA	**16**	9	8	9	9	7	13	10	8	8	9	9	**9**
**Grand Total**	**3927**	**2219**	**2286**	**2447**	**2655**	**2144**	**2630**	**2491**	**2446**	**2237**	**2485**	**2607**	**2423**

Supplementary figure 1. Composition of ncRNAdb

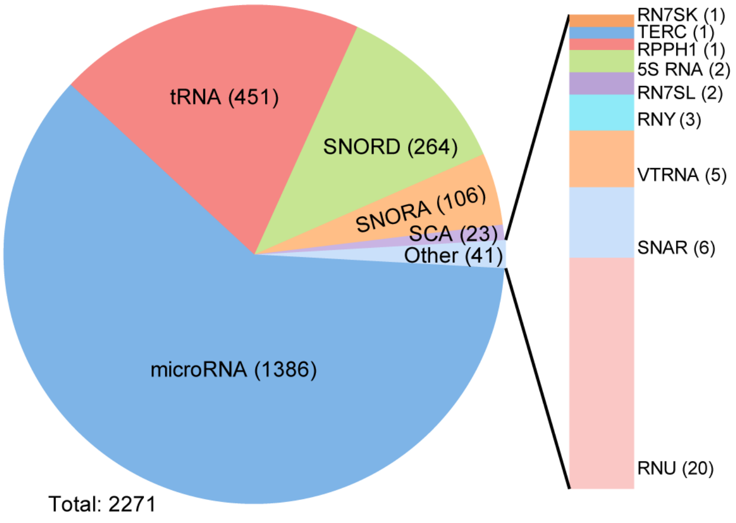

Supplementary figure 2. Total number of mapped reads per small ncRNA precursor

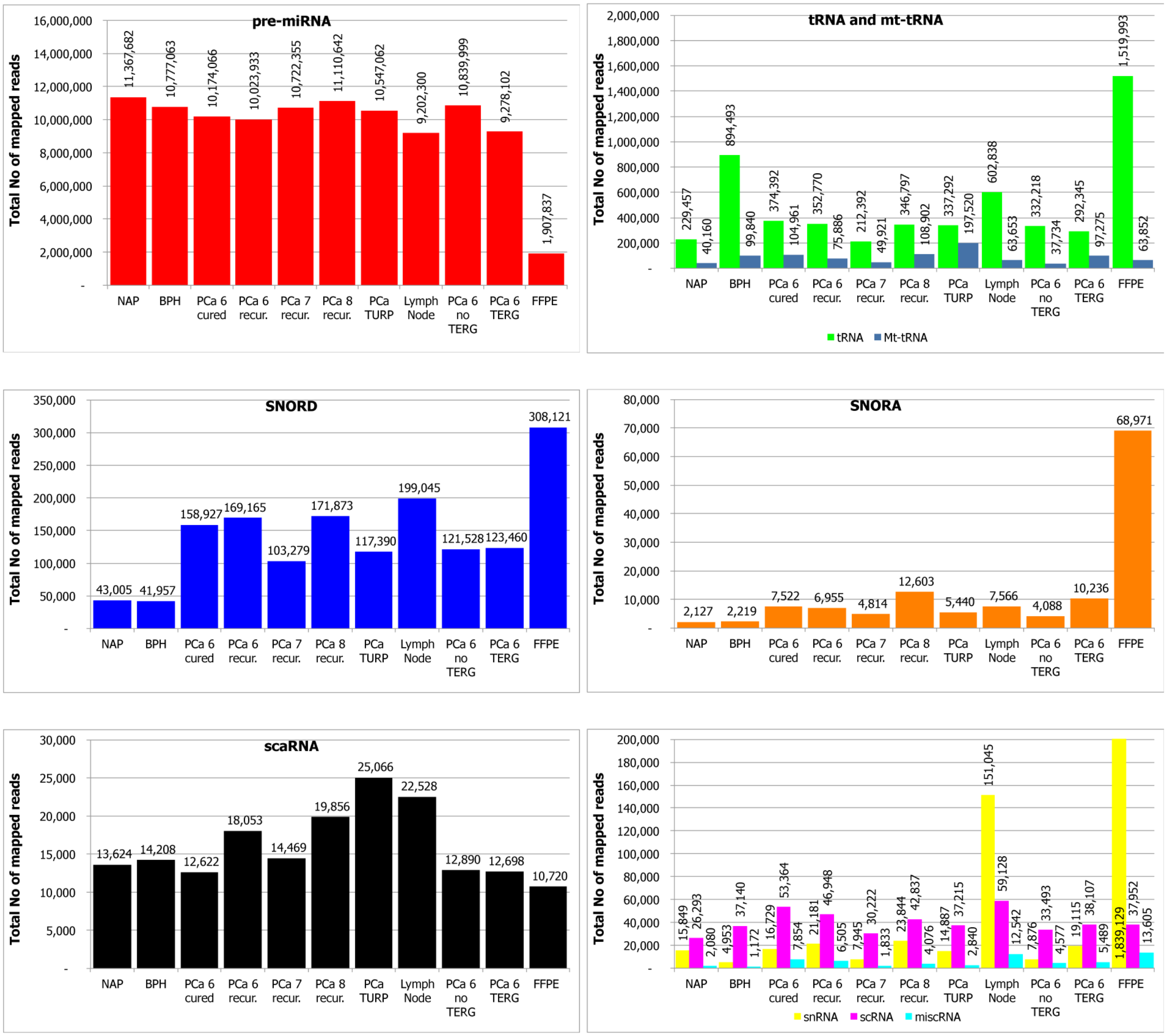

Supplementary figure 3. Size distribution of sequence reads mapping to small ncRNA in normal and malignant fresh-frozen and FFPE preserved material.

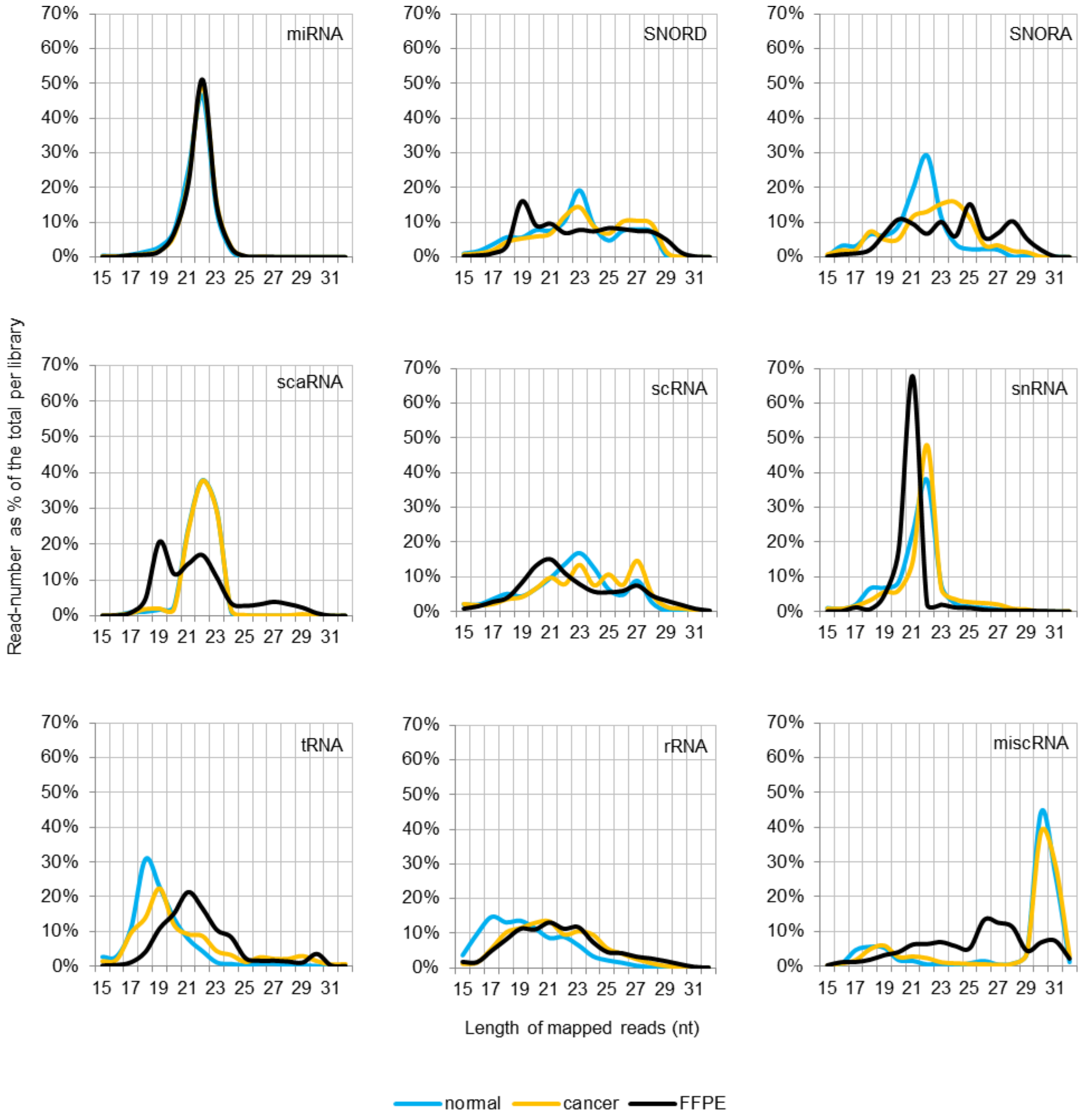

Supplementary figure 4. Validation of FlaiMapper performance. FlaiMapper predicted 5’- and 3’-ends of 707 miRNAs originating from the 592 detected in fresh frozen samples pre-miRNAs were compared with the 5’- and 3’-end boundaries of corresponding mature miRNAs in MiRBase, v.17 (ref. [1]).
The vertical axis gives the percentage of 5’-, or 3’-ends mapping to the corresponding miRBase-annotated miRNA sequence end. Exact matches are plotted at position 0 (horizontal axis). The percentages of miRNAs for which FlaiMapper detected longer or shorter ends are plotted according to the number of additional nucleotides at positions 1 to 5 and -1 to -5, respectively.

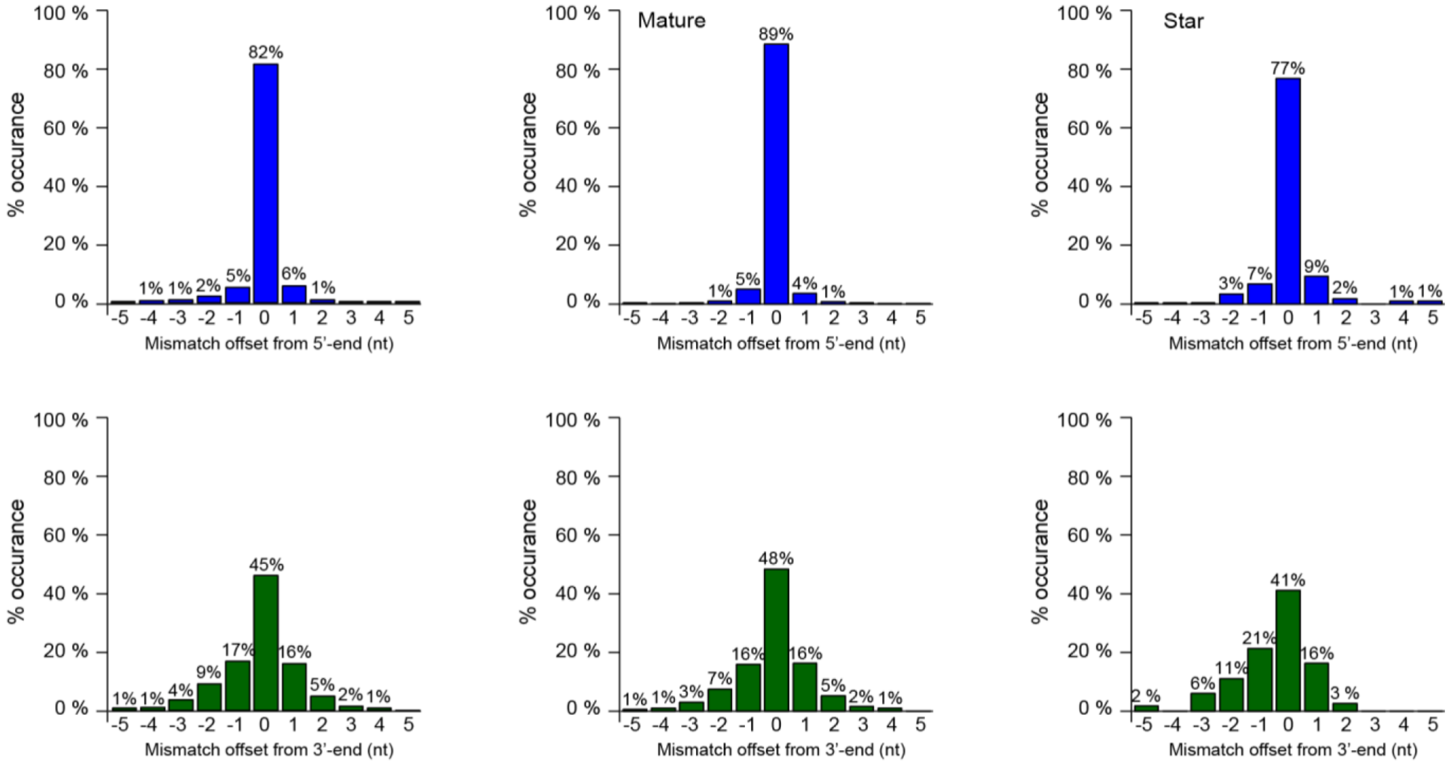

1 Kozomara A, Griffiths-Jones S. miRBase: integrating microRNA annotation and deep-sequencing data. *Nucleic Acids Res* 2011; **39**: D152-157.

Supplementary figure 5. a) Total number of detected sncdRNA precursor (left panel) and sncdRNAs (right panel per precursor type and sequencing library b) Number of unique sncdRNAs derived from individual small ncRNAs. RNU, spliceosomal RNA; RNY, Ro-associated Y RNA; VRNA, Vault RNAs; TERC, telomerase RNA component; RPPH1, ribonuclease P RNA component H1; RN7SL, signal recognition particle RNA; RN7SK, 7SK small nuclear RNA associated with P-TEFb; SNAR, small ILF3/NF90-associated RNA.

a

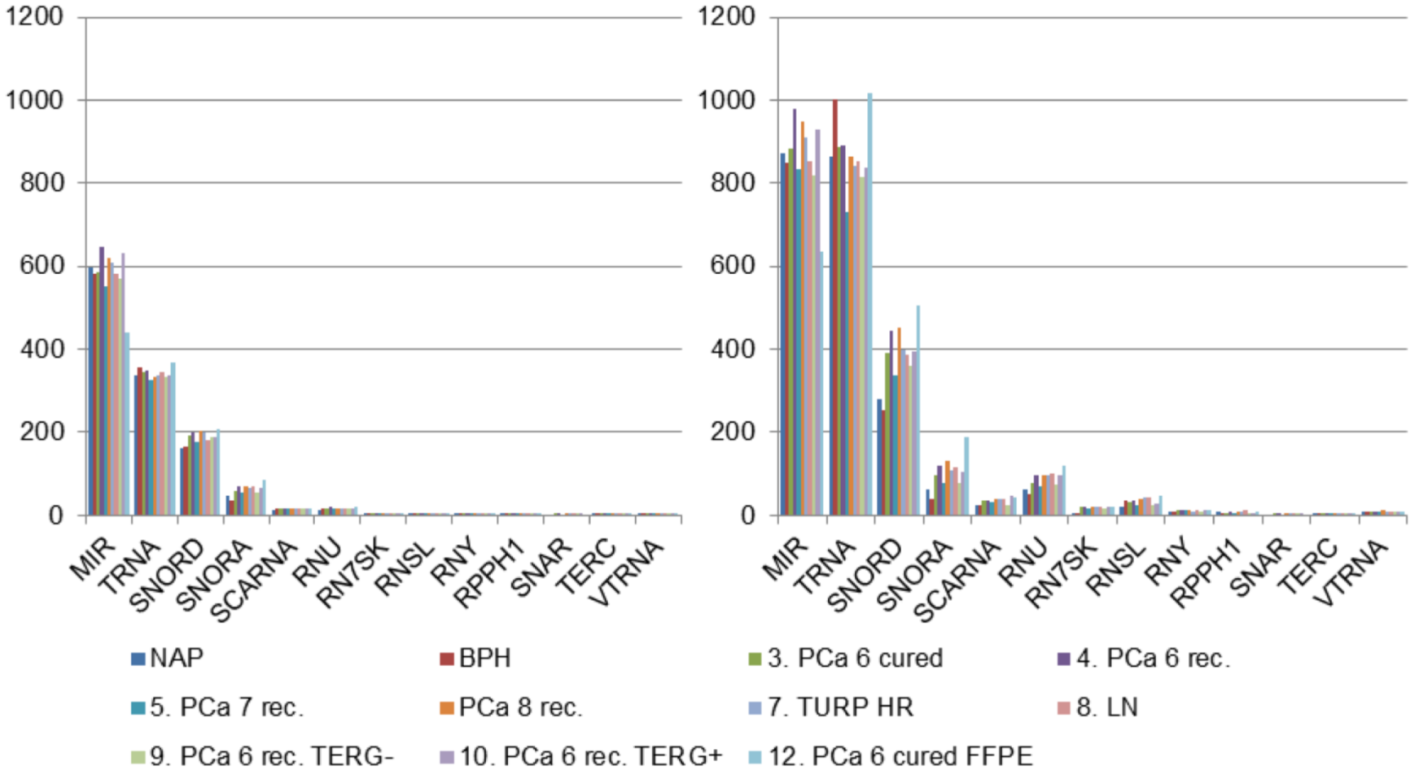

b

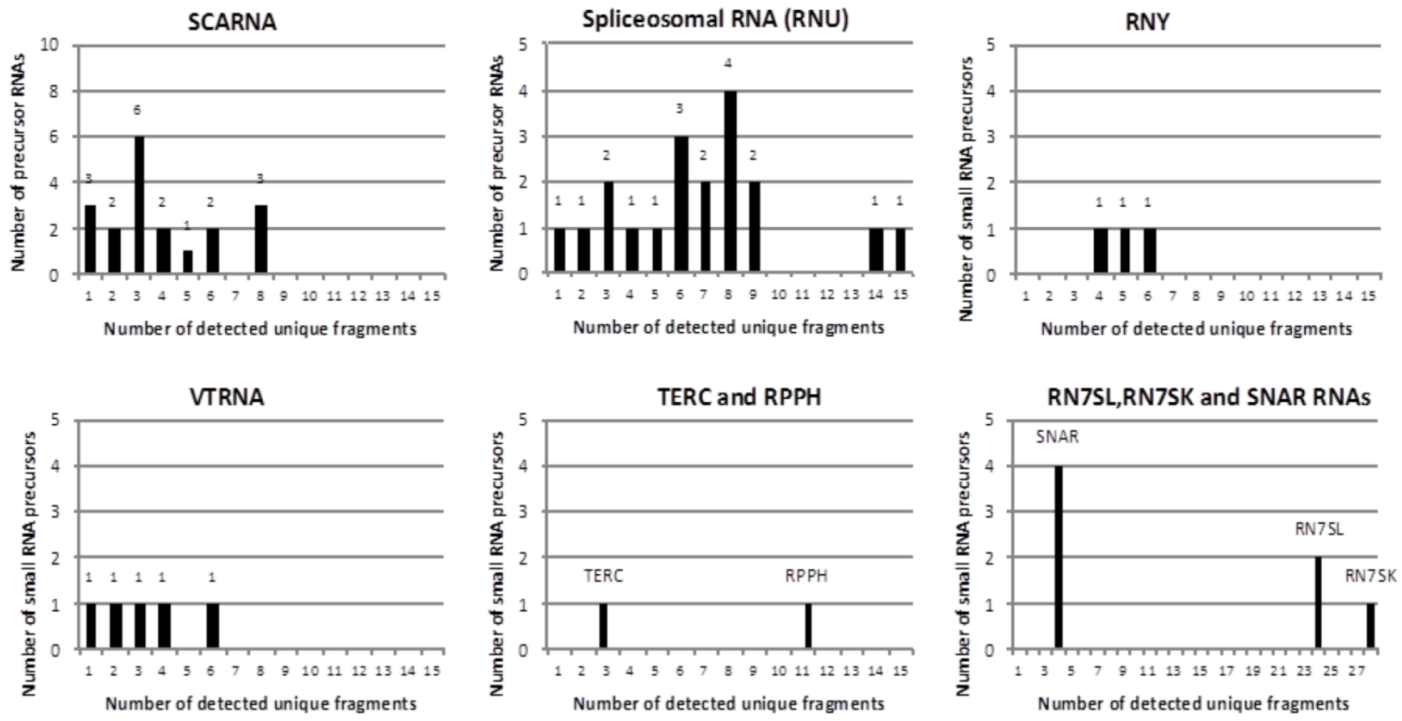

Supplementary figure 6. Length-distribution of unique sncdRNAs determined by FlaiMapper in each sequencing library. The summed number of unique sncdRNAs with a distinct length originating from a specific type of small ncRNA is plotted on the Y-axis as a percentage of the total number of unique sncdRNAs originating from that type of small ncRNA in each sequenced library.

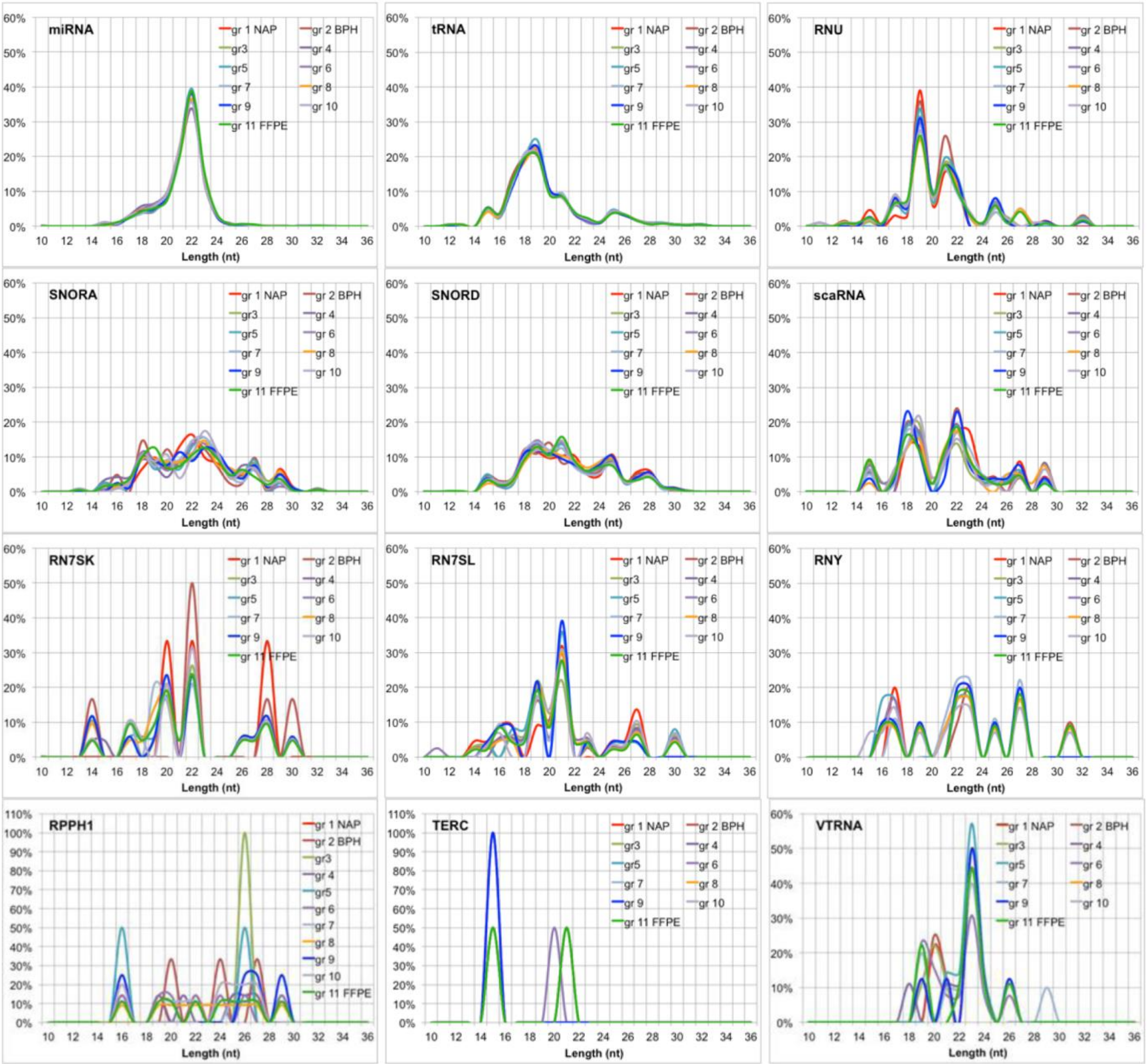

Summed number of sncdRNAs originating from each precursor corresponding to 100%

	MIR	TRNA	SNORD	SNORA	SCARNA	RNU	RN7SK	RNSL	RNY	RPPH1	SNAR	TERC	VTRNA	Total per group
**gr1**	872	866	280	61	23	64	3	22	10	7	0	2	9	**2219**
**gr2**	850	1000	255	41	25	50	6	37	10	3	0	1	8	**2286**
**gr3**	885	886	391	97	36	76	19	32	12	1	2	1	9	**2447**
**gr4**	980	891	443	119	36	98	21	36	12	7	1	2	9	**2655**
**gr5**	832	732	336	77	32	71	17	25	12	2	0	1	7	**2144**
**gr6**	948	863	453	131	40	97	21	40	12	7	3	2	13	**2630**
**gr7**	911	843	400	109	40	97	19	42	9	9	1	1	10	**2491**
**gr8**	852	852	387	116	40	100	21	43	12	11	3	1	8	**2446**
**gr9**	818	816	359	79	26	74	17	23	10	4	2	1	8	**2237**
**gr10**	928	837	394	103	46	98	19	29	14	5	1	2	9	**2485**
**gr11**	634	1017	506	189	43	119	21	47	11	9		2	9	**2607**

Supplementary figure 7. Relative abundance of sncdRNAs with different length. The relative abundance (sum of expression values) of sncdRNAs with a distinct length originating from a specific type of small ncRNA is plotted on the Y-axis as a percentage of the summed expression of all sncdRNAs originating from that type of small ncRNA in each sequencing library.

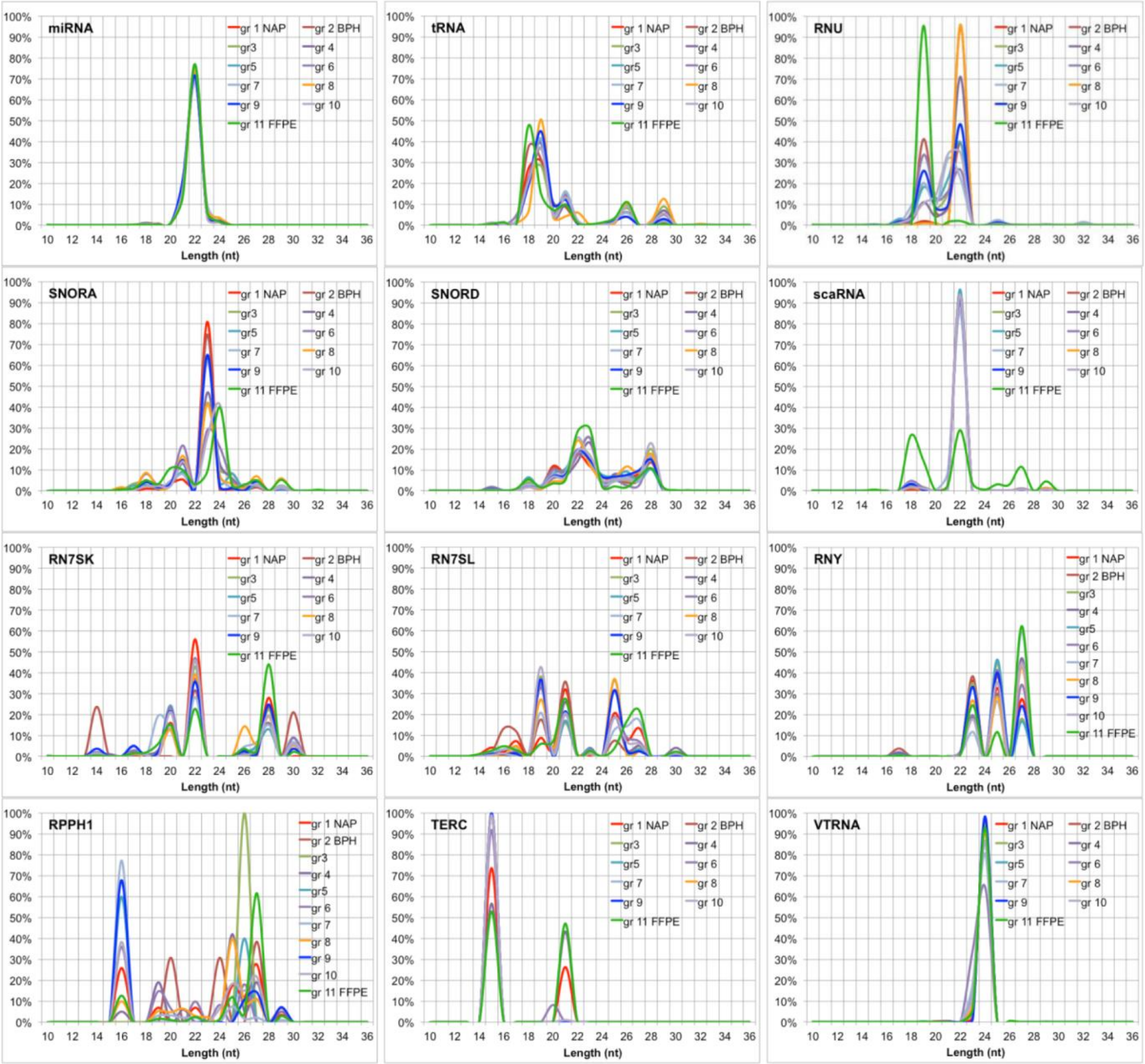

Summed expression of sncdRNAs originating from each precursor corresponding to 100%

	MIR	RN7SK	RN7SL	RNU	RNY	RPPH1	SCARNA	SNORA	SNORD	TERC	TRNA	VTRNA	Total per group
**gr1**	11 283 960	25	194	10 805	21 111	58	13 190	4 651	36 306	19	174 186	2 038	**11 546 543**
**gr2**	10 714 251	38	524	1482	30 396	13	14 296	2 842	36 241	14	770 118	3 425	**11 573 640**
**gr3**	10 084 596	2 099	784	6099	40 152	4	12 492	8 277	144 413	12	249 622	22 626	**10 571 176**
**gr4**	9 954 116	1 331	800	13 554	35 231	81	18 621	8 315	157 886	30	243 321	18 583	**10 451 869**
**gr5**	10 643 298	403	424	2 275	21 809	15	14 155	7 354	93 358	10	143 655	4 269	**10 931 025**
**gr6**	11 041 835	3 085	2 351	7 044	24 537	61	20 026	11 465	157 647	49	209 809	15 121	**11 493 030**
**gr7**	10 476 280	134	1 143	5 437	26 492	189	24 771	7 596	107 036	13	253 424	4 762	**10 907 277**
**gr8**	9 125 650	1 901	1 527	135 742	38 842	171	21 998	8 021	182 988	47	489 084	8 010	**10 013 981**
**gr9**	10 760 596	557	635	2 598	22 354	28	12 346	6 541	110 517	55	246 353	15 512	**11 178 092**
**gr10**	9 222 102	2 522	986	8 543	25 806	60	12 522	10 447	113 619	515	201 637	10 462	**9 609 221**
**gr11**	1 857 603	1 900	5 222	1 723 358	20 555	2 558	9 974	49 866	274 521	176	1 467 826	6 250	**5 419 809**

Supplementary figure 8. Differential expression of miRNAs and sncdRNAs between fresh frozen (FF) and FFPE samples.

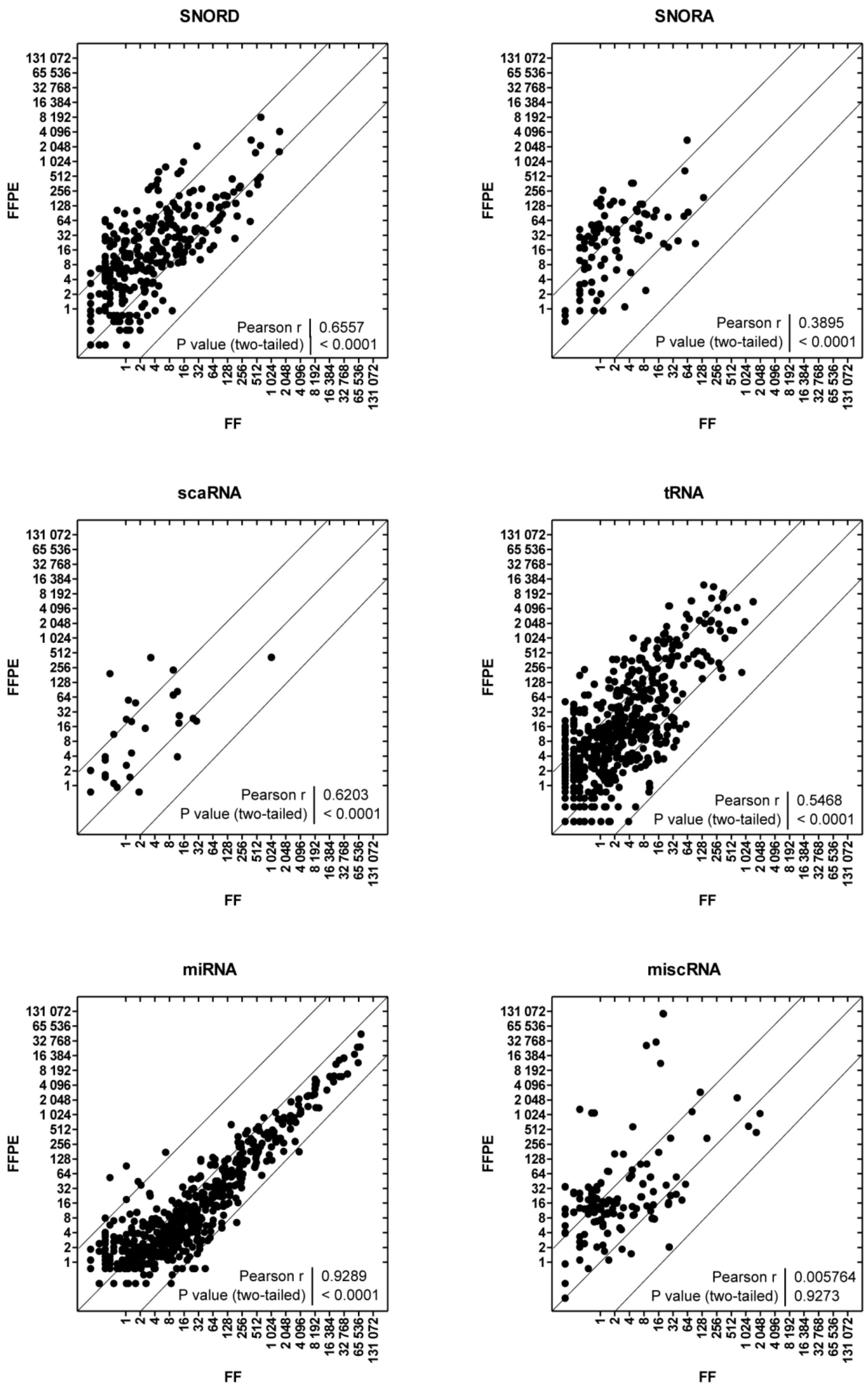

Supplementary figure 9. a) Expression of sdRNAs originating from CD-box snoRNAs encoded in the introns of the GAS5 gene from RNA-Seq data. b) Expression of full length SNORD74, SNORD44, SNORD78, SNORD81 and their sdRNAs measured by Quantitative RT-PCR with custom LNA primers.

a

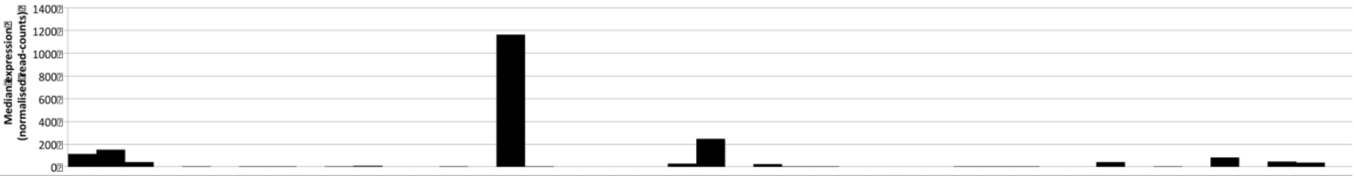

	SNORD74 Fragment_2	SNORD74 Fragment_1	SNORD74 Fragment_3	SNORD75 Fragment_2	SNORD75 Fragment_4	SNORD75 Fragment_3	SNORD75 Fragment_1	SNORD76 Fragment_1	SNORD76 Fragment_2	SNORD77 Fragment_2	SNORD77 Fragment_1	SNORD44 Fragment_1	SNORD44 Fragment_2	SNORD44 Fragment_3	SNORD44 Fragment_4
position relative to extended precursor sequence	13..37	41..61	50..76	11..29	24..41	32..54	43..70	13..30	49..68	20..35	45..62	11..38	30..50	53..71	62..82
Group 1	37	29	11	0	0	0	0	1	2	0	1	215	0	0	0
Group 2	39	15	3	0	0	0	0	0	3	0	2	282	0	0	0
Group 3	124	182	57	0	0	0	5	3	9	0	7	1497	1	0	0
Group 4	161	108	46	1	1	0	2	6	12	0	4	1280	0	0	0
Group 5	99	159	35	1	0	0	0	2	8	0	1	640	0	0	0
Group 6	117	132	26	1	0	2	4	17	10	0	13	1627	2	1	0
Group 7	83	176	33	1	0	1	1	7	4	0	2	1190	0	0	0
Group 8	202	210	120	0	0	0	8	5	7	0	2	1141	1	0	0
Group 9	109	184	44	2	0	1	0	4	6	0	7	752	0	0	0
Group 10	168	135	40	1	0	0	1	4	5	1	1	1445	1	1	0
Group 11	136	27	131	11	1	2	631	42	1	0	0	1619	11	0	0

	SNORD78 Fragment_3	SNORD78 Fragment_2	SNORD78 Fragment_1	SNORD79 Fragment_1	SNORD79 Fragment_3	SNORD79 Fragment_2	SNORD79 Fragment_5	SNORD79 Fragment_4	SNORD79 Fragment_6	SNORD80 Fragment_1	SNORD80 Fragment_4	SNORD80 Fragment_2	SNORD80 Fragment_3	SNORD47 Fragment_1	SNORD47 Fragment_3	SNORD47 Fragment_2	SNORD81 Fragment_1	SNORD81 Fragment_4	SNORD81 Fragment_3	SNORD81 Fragment_2	SNORD81 Fragment_5
position relative to extended precursor sequence	19..37	34..64	45..72	15..33	28..54	38..60	55..75	62..90	67..81	13..31	36..63	43..58	56..80	1..18	23..46	60..76	15..34	35..56	47..68	55..80	75..91
Group 1	0	3	45	2	0	0	0	0	0	0	1	0	1	15	0	0	5	0	17	4	0
Group 2	0	4	13	4	0	0	0	0	0	0	0	0	0	5	0	1	11	0	8	6	0
Group 3	1	29	487	13	0	0	0	0	0	0	0	0	0	37	0	4	86	0	66	39	0
Group 4	0	41	288	26	0	0	1	0	0	0	0	0	0	31	0	4	84	0	28	72	0
Group 5	0	12	55	6	0	0	0	0	0	1	0	0	0	45	0	2	45	0	68	32	0
Group 6	0	39	229	24	1	3	0	0	0	0	0	0	0	62	0	28	162	0	52	22	0
Group 7	0	23	223	46	1	1	0	0	0	1	0	0	1	72	0	2	148	0	28	27	0
Group 8	1	37	1083	19	5	13	0	1	0	0	1	1	0	99	1	1	77	0	83	111	0
Group 9	0	24	260	20	1	1	0	0	0	0	0	0	0	46	0	0	80	0	58	48	0
Group 10	0	37	605	30	0	0	0	0	0	0	0	0	0	19	0	3	57	0	32	46	0
Group 11	0	45	1547	19	3	2	0	8	0	29	2	2	11	281	0	60	116	3	20	39	0

0 – 1700

b

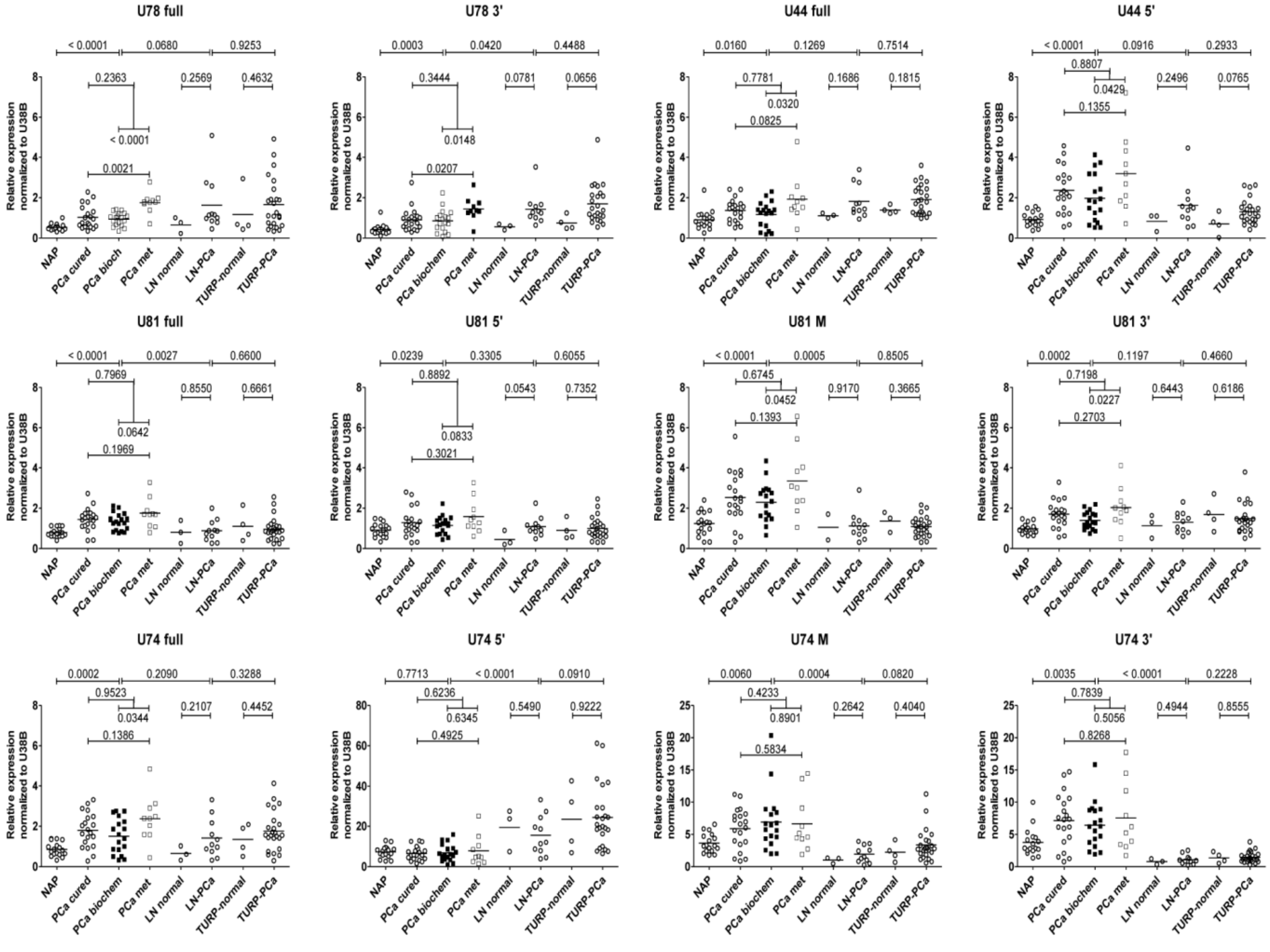

Supplementary figure 10. Overview of primary alignments of sequence reeds mapping to SNORD74, SNORD44, SNORD78, and SNORD81. Each line represents a unique sequence read. Note the different fragmentation patterns of U74 and U81 compared to U44 and U78.

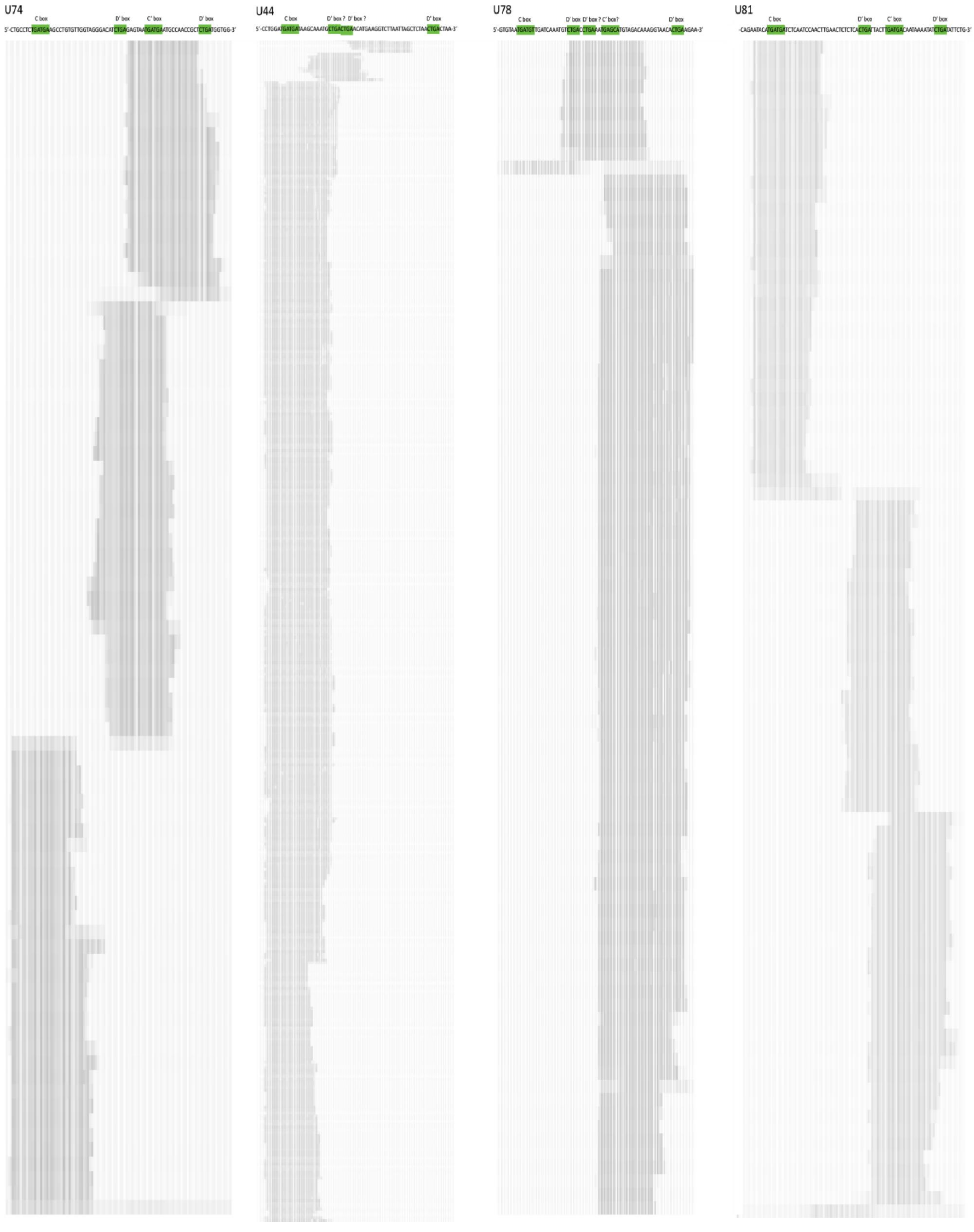

Supplementary figure 11. Expression of sd78-3', SNORD78, and GAS5 in prostate cancer cell lines and cell lines derived from normal basal prostate epithelium. Expression of sd78-3' and SNORD78 was measured using custom LNA primer assays listed in Supplementary methods table M1 and standard LNA qPCR kits (Exiqon, Copenhagen, Denmark) according to the manufacturers instructions. Expression values of sd78-3' and SNORD78 were normalized the expression of SNORD38B reference small RNA measured with Reference gene primer set 20391 (Exiqon, Copenhagen, Denmark). Spliced GAS5 transcript expression levels were assessed by the Reverse Transcription System (Promega Benelux, The Netherlands) followed by SybrGreen qPCR (Roche, The Netherlands) according to manufacturer protocols. Primers used were GAS5 FW: CAAGGACTCAGAATTCATGAT and GAS5 REV: AGTGGTCTTTGTAGACTGCC. Raw expression values were normalized against the geometrical mean of GAPDH and PBGD.

Data represents the average expression of tested RNAs in two independent biological replicates of non-consecutive cell passages. Error bars represent standard deviation.

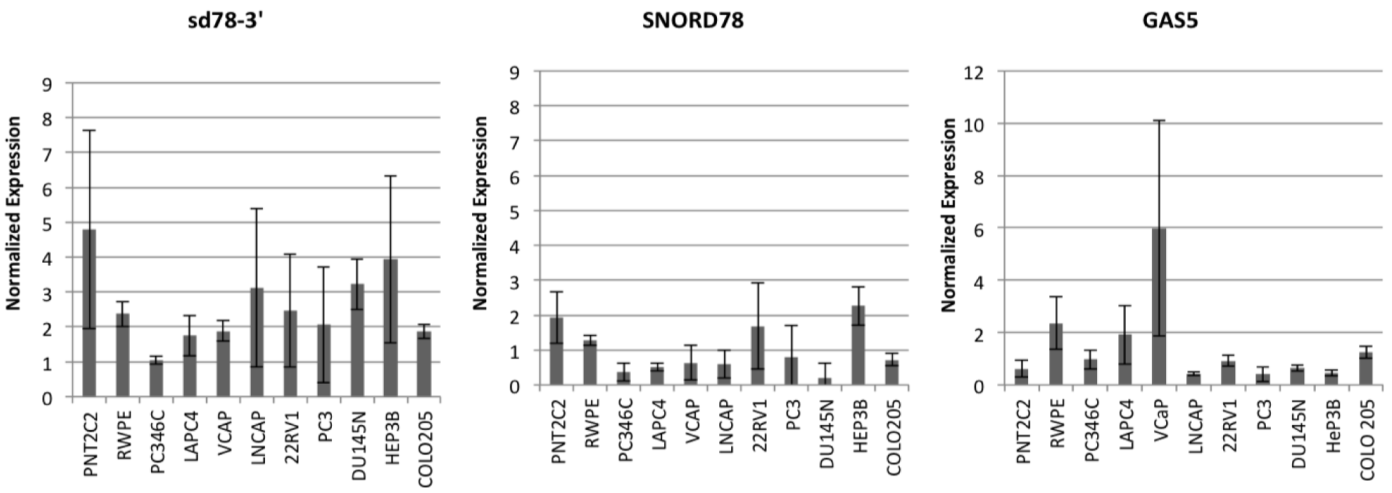

Supplement: Supplementary file 1 [file oncotarget-06-17430-s001.pdf]
