# Supplementary figures and images for: C/D-box snoRNA-derived RNA production is associated with malignant transformation and metastatic progression in prostate cancer
Source: Oncotarget. 2015 May 19;6(19):17430–44. doi: 10.18632/oncotarget.4172 (PMC4627319; doi:10.18632/oncotarget.4172)

## seq-read positions of C/D-box sdRNAs

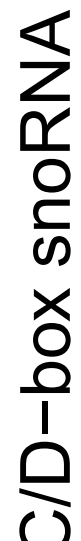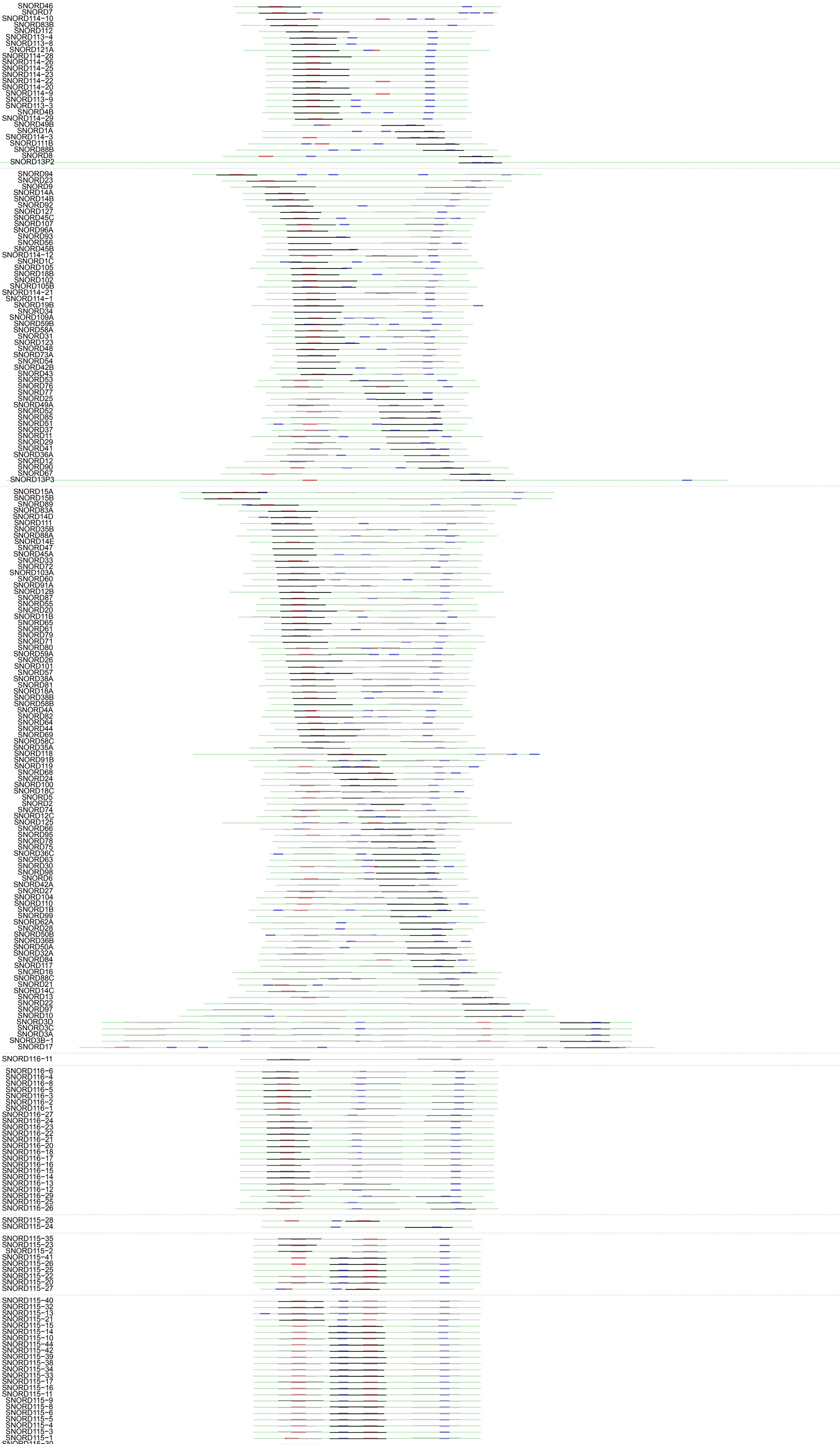

-200

-100

0

100

200

## Fixed position in nucleotides of the start of the read

Supplement: Supplementary file 5 [file oncotarget-06-17430-s005.pdf]

seq-read positions of H/ACA-box sdRNAs

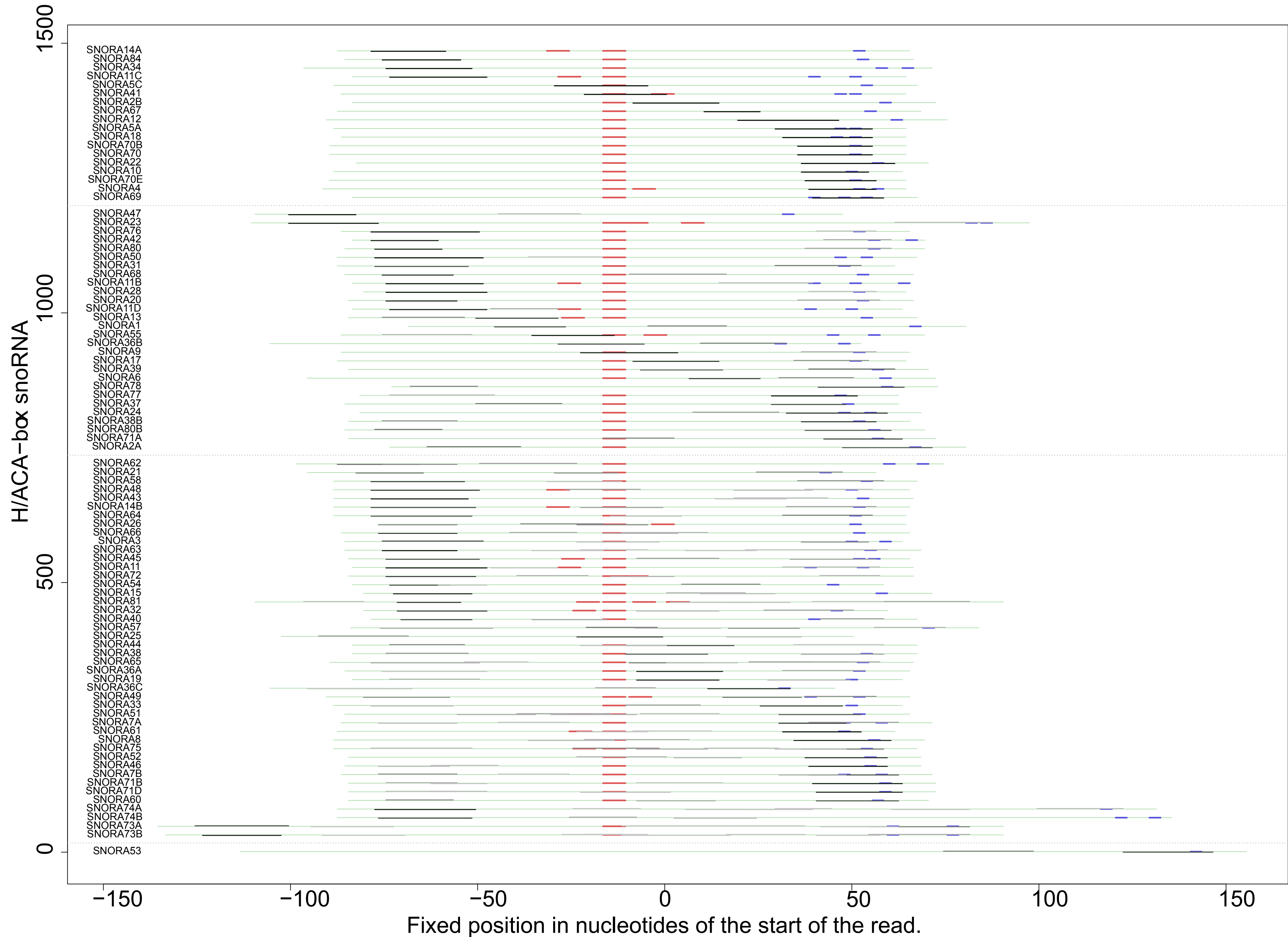

Supplement: Supplementary file 6 [file oncotarget-06-17430-s006.pdf]
